# Performance Analysis of the IEEE 802.11p Multichannel MAC Protocol in Vehicular Ad Hoc Networks

**DOI:** 10.3390/s17122890

**Published:** 2017-12-12

**Authors:** Caixia Song

**Affiliations:** College of Science and Information, Qingdao Agricultural University, Qingdao 266109, China; cassiesong@mail.dlut.edu.cn; Tel.: +86-532-8608-0444

**Keywords:** Vehicular Ad Hoc Networks (VANETs), Multichannel Medium Access Control (MAC) protocol, IEEE 1609.4 standard, Markov chain, M/G/1 queuing model, reliability, efficiency

## Abstract

Vehicular Ad Hoc Networks (VANETs) employ multichannel to provide a variety of safety and non-safety applications, based on the IEEE 802.11p and IEEE 1609.4 protocols. The safety applications require timely and reliable transmissions, while the non-safety applications require efficient and high throughput. In the IEEE 1609.4 protocol, operating interval is divided into alternating Control Channel (CCH) interval and Service Channel (SCH) interval with an identical length. During the CCH interval, nodes transmit safety-related messages and control messages, and Enhanced Distributed Channel Access (EDCA) mechanism is employed to allow four Access Categories (ACs) within a station with different priorities according to their criticality for the vehicle’s safety. During the SCH interval, the non-safety massages are transmitted. An analytical model is proposed in this paper to evaluate performance, reliability and efficiency of the IEEE 802.11p and IEEE 1609.4 protocols. The proposed model improves the existing work by taking serval aspects and the character of multichannel switching into design consideration. Extensive performance evaluations based on analysis and simulation help to validate the accuracy of the proposed model and analyze the capabilities and limitations of the IEEE 802.11p and IEEE 1609.4 protocols, and enhancement suggestions are given.

## 1. Introduction

Vehicular Ad Hoc Networks (VANETs) serve as one of the most important wireless communication technologies to implement all kinds of applications related to vehicles, road traffic, drivers, passengers and pedestrians [1]. It is considered that VANETs play an important part of the Intelligent Transportation System (ITS). VANETs consist of a set of vehicles equipped with communication devices, called On Board Units (OBUs), and a set of stationary units along the roads, called Road Side Units (RSUs). Through Vehicle To Vehicle (V2V) and Vehicle To Infrastructure (V2I) communications, vehicles can exchange information to support safety applications, such as emergency brake, cooperative collision avoidance, and automatic notification of crash on roads, and non-safety applications, such as infotainment, Internet access, video streaming, etc. [1,2,3].

**Figure 1:**
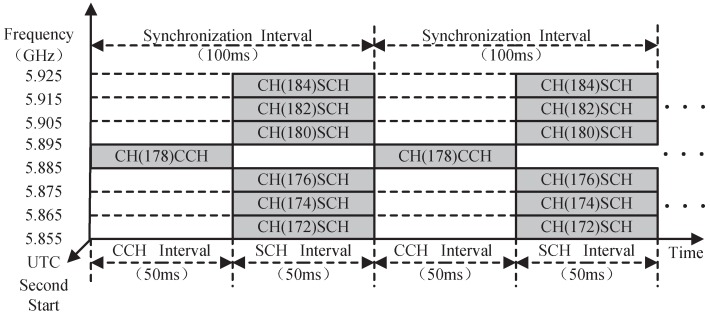
Frequency channel layout of a 5.9-GHz WAVE system.

IEEE 802.11p protocol [4] has been ratified as a standard to provide Wireless Access in Vehicular Environments (WAVE), which is based on the prioritized Enhanced Distributed Channel Access (EDCA) [5], and can support kinds of ITS applications by providing different level Quality of Service (QoS). The US Federal Communication Commission (FCC) has allocated 75 MHz in the 5.9 GHz band for Dedicated Short Range Communications (DSRC) to be used exclusively for V2V and V2I communications. The overall bandwidth is divided into seven 10-MHz channels, as shown in Figure 1. They include one Control Channel (CCH) for transmitting safety-related messages (e.g., emergency messages and periodic beacon messages) and control messages such as WAVE Service Advertisement (WSA), and six Service Channels (SCHs) for non-safety applications (e.g., comfort and infotainment). IEEE 1609.4 protocol [6] extends Medium Access Control (MAC) layer operation of the IEEE 802.11p and defines a channel switching mechanism to enable to operate efficiently on seven DSRC channels. According to the IEEE 1609.4 protocol, nodes alternately perform the transmissions of safety-related messages on one CCH and the transmissions of non-safety messages on six SCHs during the fixed CCH Interval (CCHI) and SCH Interval (SCHI), respectively. Therefore, based on multichannel architecture, both safety-related and non-safety applications can be supported [7,8].

On the CCH, each vehicle broadcasts two kinds of safety-related messages: periodic beacon and emergency (event-driven) message. Vehicles periodically broadcast beacons to perform cooperative vehicle collision avoidance such as cooperative collision warning and lane change warning [9], and under dangerous situations such as car accidents or emergency brake, the emergency messages will be triggered to disseminate to neighboring vehicles. In addition, the control messages such as WSA messages are also disseminated on the CCH, which are used to perform negotiations and reservations of the SCHs. On the other hand, the non-safety messages are transmitted on the SCHs. The traffic of safety messages has stringent requirements on highly reliable and real-time transmissions, while the non-safety applications require efficient and high throughput. Due to high vehicle mobility, ever-changing vehicle density and the requirements of different level QoS, the alternating feature of multichannel operation specified in the IEEE 1609.4 protocol leads to high packet losses on the CCH and low throughput on the SCHs [10]. Based on the EDCA mechanism, messages have different Access Categories (ACs) in a node with different priorities according to degree of emergency. In this paper, we only consider two types of ACs. Each AC on the CCH is as follows [7,11,12]: (1) $AC_{0}$ (with the higher priority) concerns the safety-related messages including periodic beacons and emergency (event-driven) messages; and (2) $AC_{1}$ (with the lower priority) concerns the control messages such as WSA messages. In VANETs, the direct broadcast on the CCH is an effective method to inform the neighborhood of safety-related messages. In addition, the reliable and efficient transmissions of WSA messages, as well as the ratio between CCHI and SCHI affect the throughput and utilization of SCHs.

This paper focuses on the performance analysis of the IEEE 1609.4 multichannel MAC protocol including the broadcast of safety-related messages on the CCH and the transmission of non-safety messages on the SCHs under a VANETs environment. We enhance the existing work by taking the following aspects into model considerations.

(1) We present two Markov chain models under both unsaturated and saturated conditions: one-dimensional Markov chain model for higher priority $AC_{0}$ and two-dimensional Markov chain model for lower priority $AC_{1}$ to analyze the real-time and reliability of safety-related messages broadcast and the WSA messages unicast on the CCH, respectively. Two M/G/1 queuing models are leveraged to derive more accurate analytical results.
(2) We take virtual collision, Arbitration Inter Frame Space (AIFS) differentiation, Contention Window (CW), the retry limit, channel switch, and the difference among frame collision probability, frame failure probability, frame blocking probability, and channel busy probability into design consideration.
(3) We calculate the Packet Reception Rate (PRR) and packet transmission delay by considering the internal virtual collisions, the collisions caused by the concurrent transmissions in the carrier-sensing range and hidden terminal range, and the packet error by fading channel.
(4) We calculate the throughput of SCHs to analyze the CCH bottleneck problem and the utilization problem of SCHs, and then give the direction of design and research of multichannel in VANETs.

## 2. Related Works

VANETs are the key components for provisioning safety-critical applications and non-safety (traffic efficient and comfort) applications on the road [13]. VANETs employ multiple channels (seven) to support these services.

The transmissions of safety-related messages are employed broadcast mode on the CCH in VANETs, and there are many works studying the broadcast performance of the safety-related messages. The work in [14] explored a large parameter space to model IEEE 802.11-based 1-D VANETs, which is derived with consideration of the IEEE 802.11 Distributed Coordination Function (DCF), and to analyze the reliability performance of one-hop safety-critical broadcast services in VANETs. Hafeez et al. [15] proposed a Markov chain model to analyze the backoff procedure of beacon and emergency message, and then the probability and throughput of packets are derived and evaluated. In [16], an analytical model for performance and reliability of safety message broadcasting was proposed. The model takes into account the vehicles’ high mobility, hidden terminal problems, the transmission collisions from neighboring vehicles, and the channel fading in VANETs. The simulation and analytical results show that the model is pretty accurate in evaluating the system reliability. In [17], an analytical model for delivering DSRC safety messages within V2V communication was proposed. Two Markov chains for different priority ACs are established to analyze the delay distribution of the safety message broadcast. Yao et al. [18] proposed two Markov chains to study the performance and the reliability of the IEEE 802.11p safety communication on the CCH. Safety-related messages are classified as four ACs with different priorities based on the degree of emergency. The PRR and packet delay are detailed analysis by taking virtual collisions, AIFS differentiation, the retry limit and the difference among frame blocking probability, frame collision probability and channel busy probability into considerations.

Some works study the safety service performance in VANETs under multichannel conditions. Campolo et al. in [19,20] analyzed the performance of periodic broadcasting of beacons and WSAs in the IEEE 802.11p by joint the feature of multichannel switch and the prioritization of messages broadcast on the CCH. The model calculates packet delivery probability as a function of CW and the number of vehicles and quantitatively characterizes the negative impact of channel switching on the performance of VANETs. An analytical model on the basis of interacting semi-Markov process which incorporates the influence of multichannel operation was designed to assess both MAC and application level performance and reliability of safety messages [21]. An optimized scheduling method was proposed to avoid synchronization collision, and channel fading is considered in the model to simulate practical communication environments. The mean transmission delay, PRR and awareness probability are derived. The work in [22] introduced a stochastic model to evaluate the delivery performance of event-driven safety messages by considering the traffic differentiation, event-driven messages lifetime, channel switching and channel error. Analytical and simulation results show that the larger CCHI can bring higher the successful delivery probability of event-driven messages.

Nevertheless, all above performance analysis are modeled for only safety-related messages on CCH, and performance of non-safety messages is not discussed. In VANETs, driven by both safety concerns and commercial interests, one of the key services offered by VANETs is popular content distribution [23,24]. The interval used to transmit safety messages at each synchronization interval is a critical parameter that directly impacts and limits the SCHI for transmitting non-safety messages [24]. The unique characteristics of the VANETs, such as highly dynamic topology, ever-changing vehicle density, unstable and variable nature of wireless links, make multichannel synchronization, coordination and access particularly challenging [25]. Wang and Hassan in [26] proposed a methodology to derive the CCHI according to requirements of safety applications, and they have analyzed the share of non-safety applications as the function of safety performance requirements and traffic density. They have found that, under high density vehicular network environments, the non-safety applications may have to be severely restricted. Misic et al. [27] investigated the performance of networks built from single-channel devices in vehicular environments. Analysis and simulation results show that channel switching causes synchronization of backoff processes, which increases the frame collision probability, in particular for small sizes of contention windows. In [28], Xiong et al. studied the relationship among the number of vehicles, packets loss ratio on the CCH, throughput of SCHs, and the duration of CCHI and SCHI. Through simulation and analysis, they found that the switch operation between fixed CCHI and SCHI cannot satisfy reliability requirement of safety applications on the CCH and high throughput requirement of non-safety applications on the SCHs. They introduce a multichannel coordination algorithm to adaptively adjust the ratio between CCHI and SCHI to achieve better performance. However, these models do not consider the effect of combined virtual collisions, fading channel and hidden terminal problem on the performance of safety and non-safety services, and these parameters are the main factors affecting the performance of the these two applications.

In this paper, we propose two analytical models for the analysis of safety and non-safety service in the IEEE 802.11p multichannel MAC protocol, taking into account virtual collisions, the retry limit, channel switch, concurrent collisions, hidden terminal problem, channel fading, unsaturated conditions, and the difference among frame collision probability, frame failure probability, frame blocking probability, and channel busy probability. We derive the transmission delay and PRR of safety-related messages to analyze the timeliness and reliability of safety-related messages transmission, and derive the throughput of non-safety messages to analyze the effectiveness of non-safety messages transmission and the utilization of SCHs. The impact of virtual collisions, channel switch, concurrent collisions, hidden terminal problem, channel fading and the length of CCHI and SCHI are evaluated by provide deeper understandings on how various factors influence safety and non-safety message delivery. The simulation and analysis results can be used to guide design of adaptive multichannel protocol in VANETs. To the best of our knowledge, this is the first attempt to model the IEEE 802.11p multichannel MAC protocol to jointly analyze the transmission delay and PRR on the CCH, and the throughput and utilization of SCHs by considering the most influential factors. The detailed comparison of different models is given in Table 1.

## 3. Background

In this section, the relevant aspects of the IEEE 802.11p and IEEE 1609.4 protocols are illustrated.

### 3.1. MAC Sublayer in IEEE 802.11p

The IEEE 802.11p protocol employ EDCA mechanism in IEEE 802.11e [5] for contention-based prioritized QoS support. According to EDCA mechanism, a station (node) can implement up four ACs with different priorities corresponding to voice, video, best effort and background traffic. Each AC has an independent MAC queue entity, which can be identified by a set of distinct channel access parameters, including CW, AIFS and Arbitrary Inter-Frame Space Number (AIFSN[AC]). The AIFSN[AC] is used to determine the duration of AIFS[AC] according to

(1) AIFS[AC]=SIFS+AIFSN[AC]×σ

where AIFSN[AC] ≥2 corresponds to Distributed Inter-Frame Space (DIFS) of IEEE 802.11, and SIFS and σ represent the duration of a Short Inter-Frame Space (SIFS) and a slot time, respectively. The default EDCA parameters setting for ACs in IEEE 802.11p is shown in Table 2 where $AC_{0}$ corresponds to the highest priority and $AC_{3}$ corresponds to the lowest priority.

**Figure 2:**
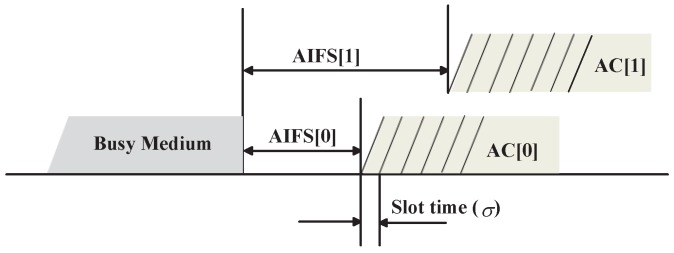
EDCA backoff procedure for two kinds of priority.

The operation of IEEE 802.11e EDCA backoff procedure described in [5] is illustrated in Figure 2. Each station has four AC queues acting as four independent stations. Note that, in this paper, we only consider two types of ACs. Each frame from the higher layer arrives at the MAC layer with a specific priority value, and enters a specific queue. The EDCA mechanism relies on the Carrier Sense Multiple Access/Collision Avoidance (CSMA/CA) technique to contend and access channel which a station must probe the channel before transmission to determine whether it is busy or idle. On one hand, if only one AC queue has backlogged data at a time in a station, and the station will sense the channel idle for the duration of AIFS[AC] as in Equation (1) before attempting to transmit it. If the channel is sensed as busy, then the station defer its transmission of an additional backoff interval. The backoff interval is calculated as a random number of slot times uniformly selected from [0, CW[AC]]. At the first transmission attempt, the backoff interval for an AC in EDCA is randomly selected from [0, CWmin[AC]], and it is doubled at every retransmission with an upper limit equal to CWmax[AC]. The smaller is AIFS[AC] or CWmin[AC], the higher is the priority in channel access. On the other hand, since each station has four different AC queues, there is a probability that more than one AC queue initiates a transmission at the same time. Therefore, an internal collision occurs inside a station, also called virtual collision. We will discuss this issue in detail in Section 4.2.

### 3.2. IEEE 1609.4 Standard

**Figure 3:**
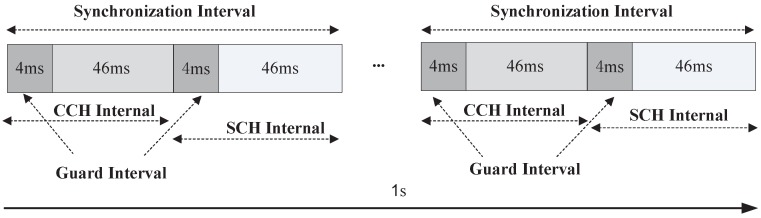
IEEE 1609.4 multichannel alternating operation.

The IEEE 1609.4 standard (protocol) is the standard of multichannel operation in VANETs. In the IEEE 1609.4 protocol, the channel time is divided into multiple Synchronization Intervals (SIs) with a fixed length of 100 ms for each SI. An SI consists of a 50 ms CCHI and a 50 ms SCHI as shown in Figure 3. Each CCHI and SCHI begins with a 4 ms guard interval which is allowed for nodes switch among channels, and it is used for precise synchronization among different nodes. All vehicles need tune to CCH during the CCHI for the transmissions of safety-related messages or WSA messages, while during the SCHI, vehicles can optionally tune to the specific SCH to deliver non-safety messages. Periodical and synchronous switching (alternating) between CCH and SCH is mandatory for single-radio transceivers and operates on one radio channel at a time.

Due to high vehicle mobility and ever-changing vehicle density, the current version of the IEEE 1609.4 standard dose not provide high QoS guarantee [7,11,30]. On the one hand, for safety-related messages, due to the mandatory channel switching and fixed duration of CCHI and SCHI specified in the IEEE 1609.4 standard, vehicular networks suffer from low reliability especially in situations of high vehicle density. On the other hand, when the traffic is sparse but heavy non-safety messages such as digital map and media downloading are required, the CCH only needs a little time to transmit a small amount of safety-related messages, and thus, the CCHI may be left idle for a significant periods of time. On the contrary, the 50 ms SCHI is not enough to transmit bulk non-safety messages. These conditions will lead to low throughput and underutilization of SCHs.

In addition, since, in the IEEE 1609.4 standard, nodes cannot specifically determine the starting time of their transmission on the SCH during the negotiation process on the CCH, there may be collisions on the SCHs. In our next analysis, we relax this condition, and assume that nodes can determine the starting time of their transmission on SCHs after their negotiation on the CCH. Base on this assume, we analyze the performance of the IEEE 1609.4 multichannel MAC protocol under a variety of vehicular environments.

## 4. Analytical Model and Performance Analysis

In this section, we propose an analytical model. The proposed analytical model takes two kinds of messages with different priorities, different packet arrival rate, unsaturated conditions, hidden terminal problem, channel switch, channel fading into account. The main symbols together with their significances used in our analytical model are given in Table 3.

To give a tractable yet reasonable model to characterize the performance of the IEEE 802.11p multichannel MAC protocol, we give some definitions and assumptions.

### 4.1. Definitions and Assumptions

**Figure 4:**
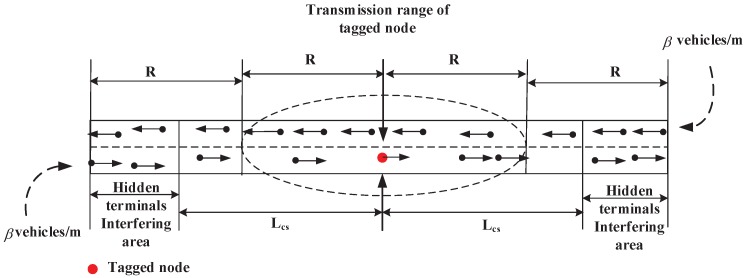
One-dimensional highway VANETs model.

In this paper, the IEEE 802.11p and IEEE 1609.4 protocols work under highway scenario with one lane in each direction, as shown in Figure 4. However, we can easily extend our analysis with the $M_{ln}$-lanes in each direction on the highway by multiplying $M_{ln}$ by the $N_{tr}$, $N_{cs}$ and $N_{int}$, respectively. Since the communication range is much larger than the road’s width, we simplify the network in each direction as one-dimensional VANETs.

The specific work environment is as follows:

**Assumption 1** **(*Poisson distribution of vehicles on road*).** *Statistic analysis of the empirical data in [31] proves that an exponential distribution is a good fit for highway vehicle traffic according to inter-vehicle distance. Assuming that the vehicle nodes are placed on the line according to a Poisson point process with network density β (in vehicles per meter), the probability of j vehicles existing within length l, P(j,l), is given by*

(2) $$P\left(j,l\right)=\frac{{\left(\beta l\right)}^{j}e^{-\beta l}}{j!}$$

**Definition 1** **(*Transmission/receiving range*).** *The transmission/receiving range R of a node represents the range within which a packet can be successfully received, if there exists no collisions from other nodes. R mainly depends on the transmission power, the data transmission rate and channel propagation characteristic. The average number of nodes within R of a tagged node, $N_{tr}$, can be given by*

(3) $$N_{tr}=4\beta R$$

**Definition 2** **(*Carrier-sensing range*).** *The carrier-sensing range $L_{cs}$ denotes the range within which a node can detect other nodes’ transmission. It is usually determined by the transmission power and antenna sensitivity. Give the tagged node is in origin, as shown in Figure 4, as long as the nodes in $\left[-L_{cs},L_{cs}\right]$ are transmitting, the tagged node will not initiate any transmission at that moment. The average number of nodes within carrier-sensing range of a tagged node, $N_{cs}$, can be given by*

(4) $$N_{cs}=4\beta L_{cs}$$

**Definition 3** **(*Interfering range/hidden terminal range*).** *The interfering range $L_{\mathrm{int}}$ is the range within which nodes in a receiving mode interfere with transmissions from other nodes. The nodes’ transmissions in $\left[-2R,-L_{cs}\right]$ and $\left[L_{cs},2R\right]$ cannot be detected by the tagged node, but interfere with the packets receiving of nodes in [−R,R] from the tagged node. The number of hidden terminal of a tagged node, $N_{\mathrm{int}}$, can be given by*

(5) $$N_{\mathrm{int}}=4\beta \left(2R-L_{cs}\right)$$

In this paper, the capture effect is not considered, and all nodes have the same *R*, $L_{cs}$ and $L_{\mathrm{int}}$.

**Assumption 2** **(*Poisson way of packet arrival rate*).** The generated packets arrive at the MAC layer in a Poisson way with rate λ. According to the IEEE 1609.4 protocol [6], the safety packets and WSA packets are sent on the CCH only during the CCHI. If the packets are generated during the SCHI, they have to wait in the MAC layer queues until the next CCHI to be transmitted. At the beginning of the next CCHI, many nodes contending the CCH, which may lead to synchronized collision [20]. To avoid synchronized collision, the considered application layer has to schedule these packets to arrive at the MAC layer queue by delaying 50 ms (a CCHI). Therefore, in this paper, the synchronized collision issue is not considered. Each node has two queues for each AC: CCHI queue and SCHI queue. CCHI queue is for the packets arriving at the MAC layer during the CCHI, and SCHI queue is for the packets arriving at the MAC layer during the SCHI. For each AC, these two queues have the same arrival rate λ. The sum of two independent Poisson processes with rate λ is the Poisson process with rate 2λ. Therefore, the packets arrival rate for safety traffic and WSA traffic during the CCHI are $2\lambda_{e}$ and $2\lambda_{s}$, respectively.

**Figure 5:**
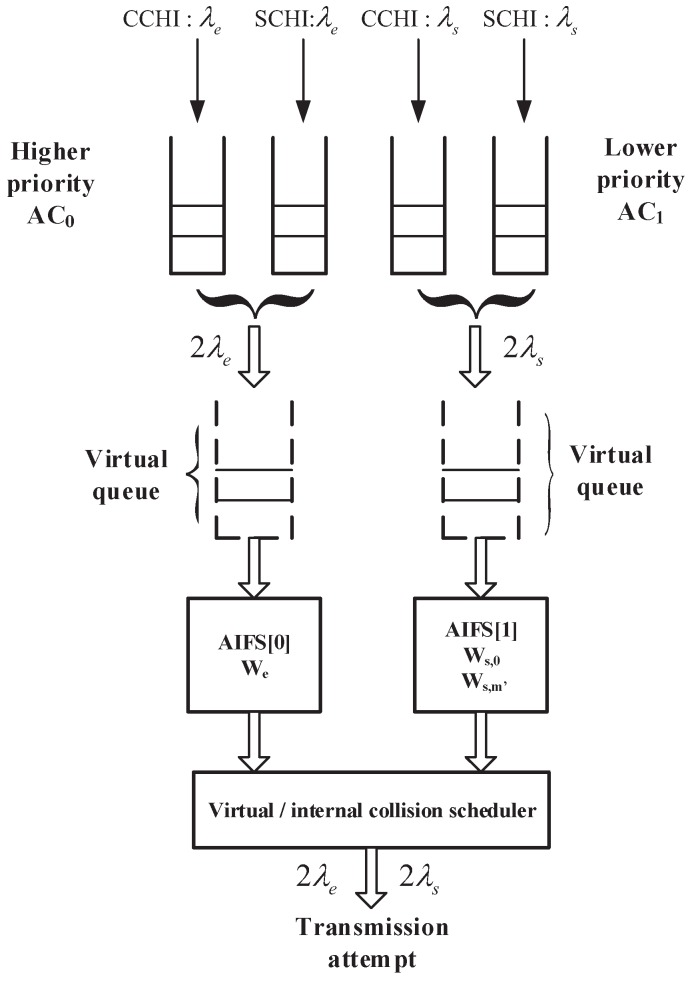
Virtual collision inside a station.

**Assumption 3** **(*Unlimited length of queue*).** Each AC has two independent queues (CCHI queue and SCHI queue) at the MAC layer with unlimited length for each, which means that the packet dropping caused by the queue overflow is not taken into consideration. These two queues can be considered one virtual queue with rate 2λ. Therefore, each node have two virtual M/G/1 queues with a common server: one virtual queue for safety packets and the other virtual queue for WSA packets, as shown in Figure 5. To simplify, the following queues refer to virtual queues.

**Assumption 4** **(*Erroneous channel Bit Error Rate (BER)*).** *In VANETs, V2V communications present environment with unfavorable characteristic of channel fading, i.e., multiple reflecting objects able to degrade the strength and quality of the received signal. In addition, high mobility makes worse Doppler spread on Orthogonal Frequency Division Multiplex (OFDM), which leads to higher BER. To simply the discuss, we follow the similar assume in [32] that control packet such as Acknowledgement (ACK) packet and frame headers (the header of both physical layer and MAC layer) of both safety and WSA packet are error free. The channel fading leads to packet error probability $P_{e,err}$ and $P_{s,err}$ for a safety packet and a WSA packet, respectively, which can be given by*

(6) $$\left\{\begin{array}{c} P_{e,err}=1-{\left(1-p_{ber}\right)}^{E[L_{e}]}\\ P_{s,err}=1-{\left(1-p_{ber}\right)}^{E[L_{s}]} \end{array}\right.$$

*where $p_{ber}$, $E[L_{e}]$ and $E[L_{s}]$ denote the probability of BER, the average payload of a safety packet and the average payload of a WSA packet, respectively. $p_{ber}$ can be numerically evaluated for a Rician fading channel [33] or Nakagami-m fading channel [34].*

**Assumption 5** **(*Neglecting the impact of vehicle mobility on the performance*).** The impact of vehicle mobility on reliability, timeliness and throughput are not considered in the model. In fact, it is illustrated in [35] that the high mobility of vehicles (up to 120 km/h) has a very small impact on the performance of the direct packet transmission with high data rate (e.g., ≥ 12 Mbps/s). In addition, the number of one-hop neighboring nodes of a tagged node does not significantly change in the short time span of our interest (a CCH interval) [36]. For example, in 50 ms, when vehicles travel in the same direction, vehicles move a very few meters forward (typically, less than 1 m in a city and less than 2 m on a highway). Even considering vehicles moving in opposite directions, for example, on a highway, vehicles travelling at 130 km/h move 3.6 m apart in 50 ms.

We also assume that transmission rate on the CCH and SCHs are constant and are the same. The non-safety messages on the SCHs have the same size, which means that all the non-safety messages occupy the same length of transmission time.

### 4.2. Virtual Collision Handing in EDCA

According to the specification in IEEE 1609.4 standard [6], each station has four AC queues (virtual queues) acting as four independent virtual stations. Each frame is mapped into an AC queue with a specified priority. When more than one AC queue (virtual queue) of a station initiates a transmission at the same time, the virtual collision occurs. The frame with highest priority is scheduled and transmitted, and the frames with lower priorities enter another backoff stage with doubled CW immediately. If the number of the retransmissions reaches the retry limit, the packet will be dropped. Note that we only discuss two kinds of packets such as safety-related packet with higher priority mapped into $AC_{0}$ queue and WSA packet with lower priority mapped into $AC_{1}$ queue on the CCH in this paper, as shown in Figure 5 . Each virtual queue corresponding to one ${\mathrm{AC}}_{i}$ (i∈[0,1]), and the packets are coming from two queues: CCHI queue and SCHI queue.

Collision may occur among different ACs in the same station (node) called virtual collision, and collision may occur among different nodes called external collision [37]. Let $P_{i,vc}$ represent the virtual collision probability of an i(i∈[e,s]) (i.e., *e* and *s* denote safety-related and WSA, respectively, and the following is the same) packet, and $P_{oc}$ represent external collision probability. Therefore, the collision probability of an i(i∈[e,s]) packet, $P_{i,c}$, can be expressed as

(7) $$P_{i,c}=P_{i,vc}+\left(1-P_{i,vc}\right)P_{oc}$$

Let $\tau_{i}$ be the internal transmission probability that the probability of transmission attempt for access class i(i∈[e,s]) in a random time slot observed by the other ACs inside a station. A virtual collision occurs if there are higher priority ACs attempt to transmit in the same station, we have

(8) $$\left\{\begin{array}{c} P_{e,vc}=0\\ P_{s,vc}=\tau_{e} \end{array}\right.$$

Let $\eta_{i}$ be the external transmission probability that the probability of transmission attempt for access class i(i∈[e,s]) observed by other stations. $\eta_{i}$ can be expressed by

(9) $$\left\{\begin{array}{c} \eta_{e}=\tau_{e}\left(1-P_{e,vc}\right)=\tau_{e}\\ \eta_{s}=\tau_{s}\left(1-P_{s,vc}\right)=\tau_{s}\left(1-\tau_{e}\right) \end{array}\right.$$

Therefore, the total transmission probability of a station, $\eta_{total}$, is calculated by

(10) $$\eta_{total}=\eta_{e}+\eta_{s}$$

**Figure 6:**
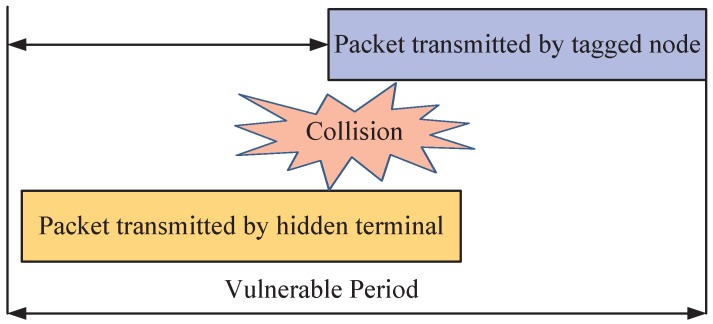
Hidden terminal vulnerable period.

For a tagged vehicle, if one of the following conditions is not satisfied, the external collision occurs: (1) when the tagged vehicle is transmitting, no vehicles within its carrier-sensing range delivery packets at the same time slot; and (2) when the tagged vehicle is transmitting, no hidden terminals start transmission during the vulnerable period $T_{vuln}$ [18,38,39], as shown in Figure 6. Each hidden terminal has chance to fail the transmission of the tagged vehicle to the target vehicle: the tagged vehicle starts sending while a hidden terminal is transmitting, or a hidden terminal starts sending while the tagged vehicle is transmitting. The vulnerable period $T_{vuln}$ is the duration that, during this period, a collision caused by hidden terminals could happen, and it is the interval from the time instance when a hidden terminal is involved in an ongoing communication before the tagged vehicle starts its transmission to the time instance when the tagged vehicle completes its transmission. Therefore, $T_{vuln}$ consists of two parts: packet transmission time of the hidden terminal and packet transmission time of the tagged vehicle. Let $T_{e}$ ($T_{s}$) and $E[L_{e}]$ ($E[L_{s}]$) denote the transmission delay and the average payload of a safety (WSA) packet, respectively, we have

(11) $$\left\{\begin{array}{cc} T_{e} & =\frac{H_{PHY}+H_{MAC}+E\left[L_{e}\right]}{R_{d}}+\delta \\ T_{s} & =\frac{H_{PHY}+E\left[L_{s}\right]}{R_{d}}+\delta \end{array}\right.$$

where $R_{d}$, $H_{MAC}$, $H_{PHY}$ and δ denote the transmission data rate on the CCH, the header length of MAC-layer, the header length of physical-layer and the propagation delay, respectively. Because there are two kinds of packets (safety-related packet with broadcast mode and WSA packet with unicast mode on the CCH), $T_{vuln}$ can be calculated by [38]:

(12) $$T_{vuln}=2\cdot \max\left(T_{e},T_{s}\right)$$

Therefore, the external collision probability $P_{oc}$ is calculated by

(13) $$\begin{array}{cc} P_{oc} & =1-\sum_{k=0}^{\infty }{\left(1-\eta_{total}\right)}^{k}\frac{{\left(N_{cs}-1\right)}^{k}}{k!}e^{-(N_{cs}-1)}\\ & \;\times {\left[\sum_{k=0}^{\infty }{\left(1-\eta_{total}\right)}^{k}\frac{N_{\mathrm{int}}^{k}}{k!}e^{-N_{\mathrm{int}}}\right]}^{T_{vuln}/T_{virt}}\\ & =1-e^{-\left(N_{cs}-1\right)\eta_{total}}e^{-N_{\mathrm{int}}\eta_{total}T_{vuln}/T_{virt}} \end{array}$$

where $T_{virt}$ denotes the average duration of a virtual time slot [40], and the expression is given in Equation (37).

### 4.3. Markov Chain for Safety-Related Message and WSA Message

**Figure 7:**
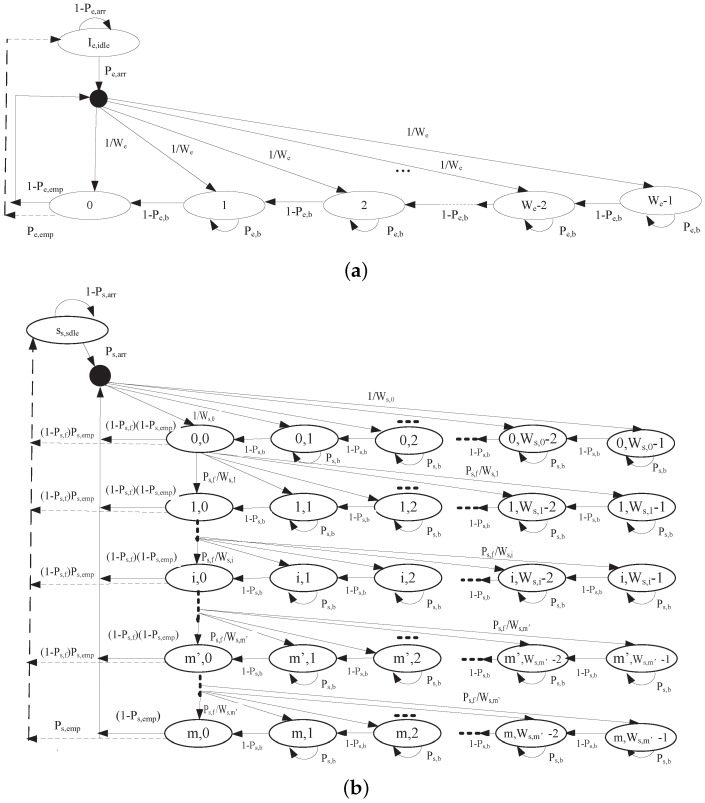
Markov chain backoff procedure: (**a**) one-dimensional Markov chain model for higher priority $AC_{0}$; and (**b**) two-dimensional Markov chain model for lower priority $AC_{1}$.

In our analytical model, each EDCA node can deliver two kinds of messages on the CCH: $AC_{0}$ and $AC_{1}$ corresponding to safety-related message and WSA message, respectively. Due to the broadcast mechanism of the transmission of the safety-related message without ACK, the sender cannot detect the external collision, and thus no retransmission will be trigged by the external collision. Therefore, the backoff procedure of safety-related message is modeled as a one-dimensional Markov chain, as shown in Figure 7a. On the other hand, the nodes use a pair of control messages such as WSA and ACK to perform negotiations on the CCH, and retransmission will be trigged due to the missing ACK. Thus, a two-dimensional Markov chain model is utilized to analyze the backoff procedure of WSA message, as shown in Figure 7b. We use Figure 7a,b to obtain the stationary internal probability $\tau_{e}$ and $\tau_{s}$, respectively. In our analytical model, unsuccessful transmission happens in two cases: (1) transmission collision due to virtual (internal) collision with the higher AC inside the same station or due to external collision with other stations; and (2) transmission error due to the unfavorable channel fading. We assume that the transmission collision or transmission error is constant and independent of the number of collisions or the number of errors of this packet has suffered in the past.

Probabilities used in two Markov chain models are listed as follows:

$P_{i,\mathrm{arr}}$: The arrival probability of i(i∈[e,s]) packets. We assume that the generated packets arrive at the MAC queues follow Poisson distributions, and the probability of receiving at least one packet in a virtual slot $T_{virt}$ is:

(14) $$P_{i,arr}=1-e^{-2\lambda_{i}T_{virt}}$$

$P_{i,\mathrm{emp}}$: The probability of i(i∈[e,s]) queue being empty. $P_{i,emp}$ can be calculated by:

(15) $$P_{i,emp}=1-\rho_{i}$$

where $\rho_{i}$ is the probability that at least one packet stays in i(i∈[e,s]) queue, and it also stands for the busy probability of server for *i* queue. $\rho_{i}$ can be derived by:

(16) $$\rho_{i}=\frac{2\lambda_{i}}{\sum_{i\in [e,s]}\mu_{i}}$$

where $\mu_{i}$ is the average service rate of virtual i(i∈[e,s]) queue (in terms of packets per second).

$P_{i,b}$: The backoff blocking probability (i.e., frame blocking probability defined in [18]). If a node senses the channel is busy due to other nodes or the other AC queues in the same node occupying the channel, the backoff timer is frozen (suspended). We have [37]

(17) $$\begin{array}{cc} P_{i,b} & =1-[\sum_{k=0}^{\infty }{\left(1-\eta_{total}\right)}^{k}\frac{{\left(N_{cs}-1\right)}^{k}}{k!}e^{-(N_{cs}-1)}\\ & \;\cdot \prod_{\begin{array}{c} j\in [e,s]\\ j\neq i \end{array}}\left(1-\tau_{j}\right){]}^{\mathrm{AIFSN}[i]-\mathrm{AIFSN}[0]+1}\\ & =1-{\left[e^{-\left(N_{cs}-1\right)\eta_{total}}\cdot \prod_{\begin{array}{c} j\in [e,s]\\ j\neq i \end{array}}\left(1-\tau_{j}\right)\right]}^{\mathrm{AIFSN}[i]-\mathrm{AIFSN}[0]+1} \end{array}$$

Note, in term AIFSN[*i*], i=e denotes AIFSN[0], and i=s denotes AIFSN[1].

$P_{i,f}$: The transmission failure probability of an i(i∈[e,s]) packet. A successful packet transmission occurs only if there no collision and no bit error during the transmission attempt. Therefore, $P_{i,f}$ can be derived as [32,41]:

(18) $$P_{i,f}=1-\left(1-P_{i,c}\right)\left(1-P_{i,err}\right)$$

Note that the interpretation of $P_{i,f}$ (i∈[e,s]) is different from *p* in [29], as it also accounts for the possibility of unsuccessful transmission caused by bit errors.

$P_{s,\mathrm{drop}}$: The probability that a WSA packet is dropped due to more than retransmission limit *m*. It can be calculated by

(19) $$P_{s,drop}=P_{s,f}^{m+1}$$

$P_{\mathrm{busy}}$: The channel busy probability. The channel sensed busy means that there is at least one node transmitting any type of packets in the carrier-sensing range $L_{cs}$. Thus, $P_{busy}$ can be calculated by

(20) $$P_{busy}=1-\sum_{k=0}^{\infty }{\left(1-\eta_{total}\right)}^{k}\frac{{N_{cs}}^{k}}{k!}e^{-N_{cs}}=1-e^{-\eta_{total}\cdot N_{cs}}$$

We analyze both one-dimensional and two-dimensional Markov chains to find the one-step transition probabilities. Let $b_{e}\left(t\right)$ denote a stochastic process representing the backoff timer value of safety traffic at time *t*. Since the transmission mode of safety packets is broadcast without backoff stages, so the fixed CW is denoted by $W_{e}$. The state of safety packet is described by $\left\{k;k\in [0,W_{e}-1]\right\}$. $I_{e,idle}$ stands for an idle state of an EDCA node with an empty safety queue. The stationary distribution of the idle state and the backoff state *k* are denoted by $b_{I_{e}}$ and $b_{e,k}$, respectively. According to Figure 7a, the one-step transition probabilities are:

(21) $$\left\{\begin{array}{c} P\left\{k|k+1\right\}=1-P_{e,b}\begin{array}{ccc} & & 0\leq k\leq W_{e}-2 \end{array}\\ P\left\{k|0\right\}=\frac{1-P_{e,emp}}{W_{e}}\begin{array}{cc} & 0\leq k\leq W_{e}-1 \end{array}\\ P\left\{k|k\right\}=P_{e,b}\begin{array}{cc} & \begin{array}{cc} & \begin{array}{cc} & 0\leq k \end{array}\leq W_{e}-1 \end{array} \end{array}\\ P\left\{I_{e,idle}|0\right\}=P_{e,emp}\\ P\left\{I_{e,idle}|I_{e,idle}\right\}=1-P_{e,arr}\\ P\left\{k|I_{e,idle}\right\}=\frac{P_{e,arr}}{W_{e}}\begin{array}{cc} & \begin{array}{ccc} & 0\leq k\leq W_{e}-1 & \end{array} \end{array} \end{array}\right.$$

The meaning of each line in Equation (21) is as follows:

(1) If the channel is idle, the backoff timer subtracts one.
(2) If the backoff timer value is zero and the queue is not empty, then the backoff timer is initially uniformly chosen in [0, $W_{e}-1$].
(3) If the channel is sensed busy, the backoff timer is frozen.
(4) If the backoff timer value is zero and the queue is empty, the node is under idle state.
(5) If no packets are to be sent in the queue and no generated packet arrives at the queue, then the node stays in the idle state.
(6) If the queue is empty, a new arriving packet makes the backoff timer uniformly chosen in [0, $W_{e}-1$].

The backoff process of WSA traffic with $AC_{1}$ is modeled as a two-dimensional Markov chain with discrete-time evolved from [7,18,29,37,41,42,43], as shown in Figure 7b. Let s(t) and b(t) be a stochastic process standing for the backoff stage and backoff timer of WSA traffic, respectively. Let the state $I_{s,idle}$ represent that the $AC_{1}$ queue of an EDCA node is empty. The maximum retransmission number and the CW in the *i*th backeoff stage are denoted by *m* and $W_{s,i}$ (i∈[0,m]), respectively. CW is set to the minimum value $W_{s,0}$ for the first transmission attempt. When collision is detected, CW is doubled and then retransmission is started for the first $m^{\prime }$ steps. While the CW remains unchanged for ($m-m^{\prime }$) steps. To sum up, if $W_{s,i}$ is the CW size in the *i*th step:

(22) $$\begin{array}{c} W_{s,i} \end{array}=\left\{\begin{array}{c} 2^{i}W_{s,0}\\ 2^{m^{\prime }}W_{s,0} \end{array}\right.\begin{array}{cc} & \begin{array}{c} \begin{array}{cc} i\leq & m^{\prime } \end{array}\\ \begin{array}{cc} m^{\prime }< & i \end{array}\leq m \end{array} \end{array}$$

Let $b_{s,i,k}=\lim_{t\to \infty }\left\{s(t)=i,b(t)=k\right\},0\leq i\leq m,0\leq k\leq W_{s,i}-1$ be the stationary distribution of the chain of WSA traffic in Figure 7b. The stationary distribution of the idle state and the backoff state are denoted by $b_{I_{s}}$ and $b_{s,i,k}$, respectively. The one-step transition probabilities are:

(23) $$\left\{\begin{array}{c} P\left\{i,k|i,k\right\}=P_{s,b}\;0\leq k\leq W_{s,i}-1,0\leq i\leq m\\ P\left\{i,k-1|i,k\right\}=1-P_{s,b}\;1\leq k\leq W_{s,i}-1,0\leq i\leq m\\ P\left\{i,k|i-1,0\right\}\;=\;\left\{\begin{array}{c} \frac{P_{s,f}}{W_{s,i}}\;0\leq k\leq W_{s,i}-1,1\leq i\leq m^{\prime }\\ \frac{P_{s,f}}{W_{s,m^{\prime }}}\;0\leq k\leq W_{s,m^{\prime }}\;-\;1,m^{\prime }\;<\;i\leq m \end{array}\right.\\ P\left\{0,k|i,0\right\}\;=\;\left\{\begin{array}{c} \frac{\left(1-P_{s,f}\right)\left(1-P_{s,emp}\right)}{W_{s,0}}\\ \;0\leq k\leq W_{s,0}-1,0\leq i\leq m-1\\ \frac{1-P_{s,emp}}{W_{s,0}}\;0\leq k\leq W_{s,0}-1,i=m \end{array}\right.\\ P\left\{0,k|I_{s,idle}\right\}=\frac{P_{s,arr}}{W_{s,0}}\;0\leq k\leq W_{s,0}-1\\ P\left\{I_{s,idle}|i,0\right\}=\left\{\begin{array}{cc} \left(1-P_{s,f}\right)P_{s,emp} & 0\leq i\leq m-1\\ P_{s,emp} & i=m \end{array}\right.\\ P\left\{I_{s,idle}|I_{s,idle}\right\}=1-P_{s,arr} \end{array}\right.$$

The meaning of each line in Equation (23) is as follows:

(1) If the channel is busy, the backoff timer is frozen.
(2) If the channel is free, the backoff timer will subtract one.
(3) Within $m^{\prime }$ backoff stage, transmission failure makes backoff stage increase and CW double. Otherwise, transmission failure makes CW remain $2^{m^{\prime }}W_{s,0}$.
(4) If a WSA packet is successfully transmitted or a WSA packet reaches its maximum retransmission number *m*, backoff stage is reset.
(5) If an EDCA node is in state $I_{s,idle}$, a new arrived WSA packet makes it enter backoff stage 0, and then node uniformly chooses backoff timer value in [0, $W_{s,0}-1$].
(6) After every successful transmission of the WSA packet or a WSA packet reaches its maximum retransmission number *m*, an EDCA node enters state $I_{s,idle}$ with a probability of $P_{s,emp}$.
(7) Without a new WSA packet arriving, an EDCA node’s idle state $I_{s,idle}$ remains unchanged.

According to the Markov chain regularities in the steady state, we derive the following relationships:

(24) $$\left\{\begin{array}{c} b_{e,k}=\frac{W_{e}-k}{W_{e}\left(1-P_{e,b}\right)}b_{e,0},\;1\leq k\leq W_{e}-1\\ b_{I_{e}}=P_{e,emp}b_{e,0}+b_{I_{e}}\left(1-P_{e,arr}\right) \end{array}\right.$$

(25) $$\left\{\begin{array}{c} b_{s,i,k}=\frac{W_{s,i}-k}{W_{s,i}\left(1-P_{s,b}\right)}b_{s,i,0}\\ \;=\left\{\begin{array}{ll} \frac{2^{i}W_{s,0}-k}{2^{i}W_{s,0}\left(1-P_{s,b}\right)}b_{s,i,0}, & 0\leq i\leq m^{\prime },1<k\leq W_{s,i}-1\\ \frac{2^{m^{\prime }}W_{s,0}-k}{2^{m^{\prime }}W_{s,0}\left(1-P_{s,b}\right)}b_{s,i,0}, & m^{\prime }<i\leq m,1<k\leq W_{s,i}-1 \end{array}\right.\\ b_{I_{s}}=\left(1-P_{s,f}\right)P_{s,emp}\sum_{i=0}^{m-1}b_{s,i,0}\\ \;+P_{s,emp}b_{s,m,0}+b_{I_{s}}\left(1-P_{s,arr}\right)\\ b_{s,i-1,0}P_{s,f}=b_{s,i,0}\to b_{s,i,0}=P_{s,f}^{i}b_{s,0,0},\begin{array}{c} 0<i\leq m \end{array} \end{array}\right.$$

According to the stationary distribution of the Markov chain, we express the total probability as:

(26) $$b_{I_{e}}+\sum_{k=0}^{W_{e}-1}b_{e,k}=1$$

(27) $$b_{I_{s}}+\sum_{i=0}^{m}\sum_{k=0}^{W_{s,i}-1}b_{s,i,k}=1$$

Using Equations (24) and (26), internal transmission probability $\tau_{e}$ of a safety-related packet in a randomly slot time is

(28) $$\tau_{e}=b_{e,0}={\left[\frac{W_{e}-1}{2(1-P_{e,b})}+\frac{P_{e,emp}+P_{e,arr}}{P_{e,arr}}\right]}^{-1}$$

Combining Equations (25) and (27), we get

(29) $$b_{s,0,0}=\left\{\begin{array}{c} \begin{array}{c} {}[\frac{W_{s,0}\left[1-{\left(2P_{s,f}\right)}^{m+1}\right]}{2\left(1-P_{s,b}\right)\left(1-2P_{s,f}\right)}-\frac{1-P_{s,f}^{m+1}}{2\left(1-P_{s,b}\right)\left(1-P_{s,f}\right)}\\ +\frac{P_{s,emp}}{P_{s,arr}}+\frac{1-P_{s,f}^{m+1}}{1-P_{s,f}}{]}^{-1},m\leq m^{\prime }\\ \left[\frac{W_{s,0}\left[1-{\left(2P_{s,f}\right)}^{m^{\prime }+1}\right]}{2\left(1-P_{s,b}\right)\left(1-2P_{s,f}\right)}\right.\\ +\frac{2^{m^{\prime }}W_{s,0}\left(P_{s,f}^{m^{\prime }+1}-P_{s,f}^{m+1}\right)+P_{s,f}^{m+1}-1}{2\left(1-P_{s,b}\right)\left(1-P_{s,f}\right)}\\ +\frac{P_{s,emp}}{P_{s,arr}}+\frac{1-P_{s,f}^{m+1}}{1-P_{s,f}}{]}^{-1},m>m^{\prime } \end{array} \end{array}\right.$$

As any transmission occurs when the backoff timer is equal to zero, regardless of the backoff stage, the internal transmission probability $\tau_{s}$ that a node transmits a WSA packet in a random time slot can be given by

(30) $$\tau_{s}=\sum_{i=0}^{m}b_{s,i,0}=\sum_{i=0}^{m}P_{s,f}^{i}b_{s,0,0}=\frac{1-P_{s,f}^{m+1}}{1-P_{s,f}}b_{s,0,0}$$

Now, we calculate **average virtual slot** $T_{\mathrm{virt}}$ [40]. Let $T_{e,suc}$ ($T_{e,col}$) and $T_{s,suc}$ ($T_{s,col}$) denote the time of successful transmission (transmission collision) of a safety packet and the time of successful reservation (transmission collision) of a WSA packet, respectively. Due to our Assumption 4 in Section 4.1, the time for transmission collision equals to the time for transmission bit error, and thus, for simplification, $T_{e,col}$ ($T_{s,col}$) also denotes the time for transmission bit error of safety (WSA) packet. We have

(31) $$T_{e,suc}=T_{e,col}=T_{e}+\mathrm{AIFS}[0]$$

(32) $$\left\{\begin{array}{c} T_{s,suc}=T_{s}+\mathrm{AIFS}[1]+SIFS+T_{ACK}+\delta \\ T_{s,col}=T_{s}+\mathrm{AIFS}[1] \end{array}\right.$$

where $L_{ACK}$ and $T_{ACK}=E\left[L_{ACK}\right]/R_{d}$ denote the packet size of an ACK packet and the time for transmitting an ACK packet, respectively.

Let $P_{e,suc}^{v}$ and $P_{s,suc}^{v}$ be the probability that the transmission attempt of an safety packet and a WSA packet are successful, respectively, which have no collisions caused by: the transmissions of higher ACs within the same station, concurrent transmissions of the other stations within the sensing-carrier range, the hidden terminal problem and the packets dropped due to more than retransmission limit for WSA packet, conditioned on the fact that at least one station transmits in the considered time slot. By definition,

(33) $$\left\{\begin{array}{c} \begin{array}{cc} P_{e,suc}^{v} & =\frac{N_{tr}\times \eta_{e}\times \sum_{k=0}^{\infty }{\left(1-\eta_{total}\right)}^{k}\frac{{\left(N_{cs}-1\right)}^{k}}{k!}e^{-(N_{cs}-1)}}{P_{busy}}\\ & \;\times {\left[\sum_{k=0}^{\infty }{\left(1-\eta_{total}\right)}^{k}\frac{N_{\mathrm{int}}^{k}}{k!}e^{-N_{\mathrm{int}}}\right]}^{T_{vuln}/T_{virt}}\\ & =\frac{N_{tr}\times \eta_{e}\times e^{-\eta_{total}\left(N_{cs}+N_{\mathrm{int}}T_{vuln}/T_{virt}-1\right)}}{P_{busy}}\\ P_{s,suc}^{v} & =\frac{N_{tr}\times \eta_{s}\times \sum_{k=0}^{\infty }{\left(1-\eta_{total}\right)}^{k}\frac{{\left(N_{cs}-1\right)}^{k}}{k!}e^{-(N_{cs}-1)}}{P_{busy}}\\ & \;\times {\left[\sum_{k=0}^{\infty }{\left(1-\eta_{total}\right)}^{k}\frac{N_{\mathrm{int}}^{k}}{k!}e^{-N_{\mathrm{int}}}\right]}^{T_{vuln}/T_{virt}}\\ & \;\times (1-P_{s,drop})\\ & =\frac{N_{tr}\times \eta_{s}\times e^{-\eta_{total}\left(N_{cs}+N_{\mathrm{int}}T_{vuln}/T_{virt}-1\right)}}{P_{busy}}\\ & \;\times (1-P_{s,drop}) \end{array} \end{array}\right.$$

Let $P_{col}$ be the probability that a transmission fails due to a collision given that there is at least one other station transmits in the considered time slot, we have [37]

(34) $$P_{col}=P_{busy}-P_{busy}\sum_{i\in [e,s]}P_{i,suc}^{v}$$

Based on our Assumption 4 in Section 4.1, there are six types of virtual slots : empty slots (all nodes are in backoff procedure or idle), safety packet error slots, successful transmitting safety packet slots, WSA packet error slots, successful transmitting WSA packet slots, collision slots from only emergency packets, only WSA packets or both, and let $T^{1}$, $T^{2}$, $T^{3}$, $T^{4}$, $T^{5}$ and $T^{6}$ be the duration, let $P^{1}$, $P^{2}$, $P^{3}$, $P^{4}$, $P^{5}$, and $P^{6}$ be the probabilities, corresponding to the above slots, respectively. Their durations and probabilities are expressed by Equations (35) and (36), respectively.

(35) $$\left\{\begin{array}{c} \begin{array}{cc} T^{1} & =\sigma \\ T^{2} & =T_{e,col}\\ T^{3} & =T_{e,suc}\\ T^{4} & =T_{s,col}\\ T^{5} & =T_{s,suc}\\ T^{6} & =\max(T_{e,col},T_{s,col}) \end{array} \end{array}\right.$$

(36) $$\left\{\begin{array}{c} \begin{array}{cc} P^{1} & =1-P_{busy}\\ P^{2} & =P_{busy}P_{e,suc}^{v}\left[1-{\left(1-p_{ber}\right)}^{E[L_{e}]}\right]\\ P^{3} & =P_{busy}P_{e,suc}^{v}{\left(1-p_{ber}\right)}^{E[L_{e}]}\\ P^{4} & =P_{busy}P_{s,suc}^{v}\left[1-{\left(1-p_{ber}\right)}^{E[L_{s}]}\right]\\ P^{5} & =P_{busy}P_{s,suc}^{v}{\left(1-p_{ber}\right)}^{E[L_{s}]}\\ P^{6} & =P_{col} \end{array} \end{array}\right.$$

Therefore, $T_{virt}$ can be expressed as

(37) $$T_{virt}=\sum_{i=1}^{6}P^{i}T^{i}$$

Using Equations (2)–(15), (17)–(20), (22) and (28)–(37), variables $\tau_{e}$, $\tau_{s}$, $P_{e,b}$, $P_{s,b}$, $P_{e,f}$ and $P_{s,f}$ can be solved by the numerical methods as in [29], where $0<\tau_{e},\tau_{s},P_{e,b},P_{s,b},P_{e,f},P_{s,f}<1$.

Our Markov chain models are derived from the fundamental developments of the models by Giuseppe Bianchi [29], and extend the Bianchi’s models to prioritized scheme by introducing multiple ACs with distinct parameters setting. Moreover, we also take into account virtual collisions, the retry limit, channel switch, channel fading, unsaturated conditions, and the difference among frame collision probability, frame failure probability, frame blocking probability, and channel busy probability.

Note that if $P_{e,emp}=1-\rho_{e}=1$ or $P_{s,emp}=1-\rho_{s}=1$, it means that there is no packet to transmit and it is the extreme unsaturated condition. This is similar to the Markov chain model proposed in [17,18] under bit error free conditions. On the other hand, if $P_{e,emp}=1-\rho_{e}=0$ or $P_{s,emp}=1-\rho_{s}=0$, it implies that there is always packet to transmit and it is the saturated condition. This is similar to the Markov chain model proposed in [37] under saturation and bit error free conditions. Thus, our model covers the extreme unsaturated, intermediate unsaturated, and saturated conditions by taking the queuing status ($P_{e,emp}$, $P_{s,emp}$) into account. The value of $P_{e,emp}$ and $P_{s,emp}$ will be derived later in the next section. Besides, our proposed analytical model considers the impact of bit error caused by fading channel which is an unfavorable characteristics of VANETs due to highly mobility of vehicles and constantly changing topology of vehicular network [44].

### 4.4. Performance Analysis

#### 4.4.1. Packet Transmission Delay

**Definition 4** **(*Packet transmission delay*).** The packet transmission delay is the time duration between the time instant that a packet arrives at the queue and the time instant that this packet is successful transmitted or dropped. There are two cases for packet dropped: on case is that for a safety packet, the packet is dropped due to expiration. The other case is that a WSA packet is dropped due to reaching the maximum retransmission number.

Due to the channel switching, each packet arriving at the MAC queue during SCHI has to wait, until the end of SCHI to be transmitted. Since the time of packets arriving at the MAC queue is random, the delay in the SCHI can be approximated by half of SCH interval. On the other hand, when a node contends for CCH (a common server) to transmit a packet during the CCHI, the average transmission delay of an *i* (i∈[e,s]) packet in the CCHI consists of service time $TS_{i}$ and queuing time $TQ_{i}$. Let $D_{e}$, $D_{s}$ and $T_{SCHI}$ denote the total transmission delay of a safety packet, the total transmission delay of a WSA packet and the duration of SCH interval, respectively, we have

(38) $$\left\{\begin{array}{cc} D_{e} & =\frac{T_{SCHI}}{2}+TS_{e}+TQ_{e}\\ D_{s} & =\frac{T_{SCHI}}{2}+TS_{s}+TQ_{s} \end{array}\right.$$

Now, we calculate $TS_{e}$ ($TS_{s}$) and $TQ_{e}$ ($TQ_{s}$).

**Figure 8:**
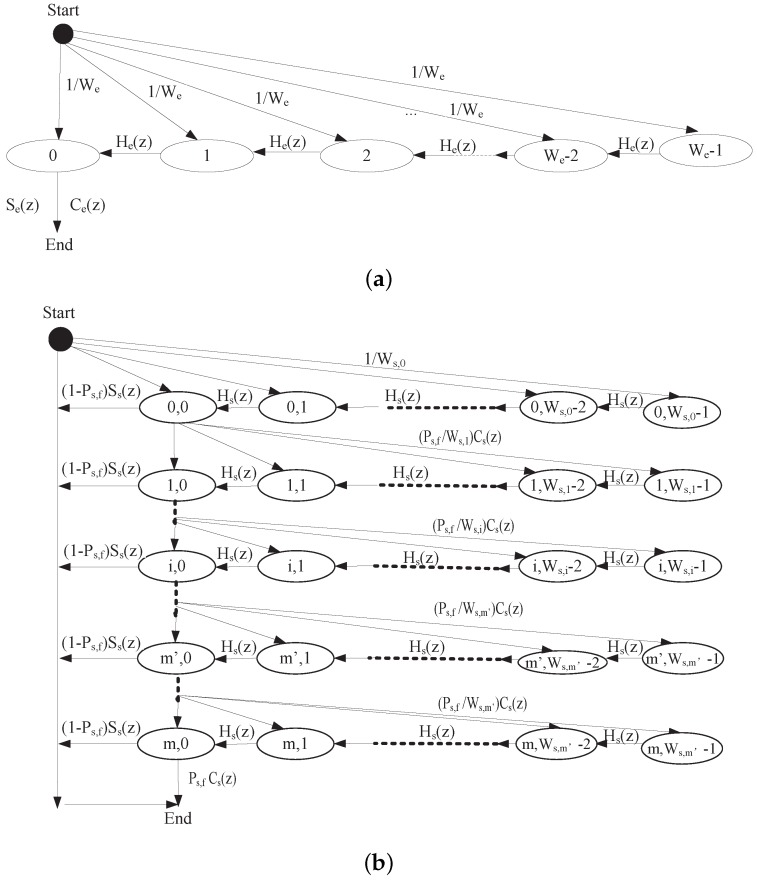
Generalized state transition diagram: (**a**) safety-related packets with higher priority $AC_{0}$; and (**b**) WSA packets with lower priority $AC_{1}$.

**Definition 5** **(*Service time*).** *The service time $TS_{i}$ is the duration from the time instant when an i (i∈[e,s]) packet becomes the head of the queue to the time instant when this packet is transmitted or dropped. The service time is important to examine the performance of higher protocol layer [39]. Since the smallest time unit of backoff timer is a time slot σ, the service time of packets is a nonnegative random variable whose distribution is a discrete probability distribution. In this paper, by following the similar approach in [39,45], we model each entity as an M/G/1 queue with unlimited length and apply the Probability Generating Function (PGF) approach to transform the Markov chain into the z domain, as shown in Figure 8. Let $q_{i,k}$ be the steady state probability of i (i∈[e,s]) packets that the packet service time is $ts_{i,k}\cdot \sigma$. Let $P_{TS_{i}}$ denote the PGF of $q_{i,k}$, which is expressed as*

(39) $$P_{TS_{i}}\left(z\right)=\sum_{k=0}^{\infty }q_{i,k}z^{ts_{i,k}}$$

The service time includes the backoff time and the transmission time. In the EDCA backoff procedure, for a safety packet, the backoff timer will be decremented by a time slot (σ) with probability (1 − $P_{e,b}$), while it is frozen for a period of time when the channel is busy. There are five cases making backoff timer frozen: safety packet error with probability $P^{2}$, successful transmission a safety packet with probability $P^{3}$, WSA packet error with probability $P^{4}$, successful transmission a WSA packet with probability $P^{5}$, collision slots from only emergency packets, only WSA packets or both with probability $P_{e,b}-P^{2}-P^{3}-P^{4}-P^{5}$. Let $S_{e}\left(z\right)$ and $C_{e}\left(z\right)$ be the PGF of $T_{e,suc}$ and $T_{e,col}$, respectively, we have

(40) $$S_{e}\left(z\right)=C_{e}\left(z\right)=z^{\left\lfloor \frac{T_{e,suc}}{\sigma }\right\rfloor }$$

where ⌊·⌋ is a function to round floating point numbers to integers. Therefore, the PGF of the time that the backoff timer of safety packet decrements by one can be expressed by

(41) $$\begin{array}{cc} H_{e}\left(z\right) & =\left(1-P_{e,b}\right)z+P^{2}C_{e}\left(z\right)+P^{3}S_{e}\left(z\right)+P^{4}C_{s}\left(z\right)\\ & \;+P^{5}S_{s}\left(z\right)+\left(P_{e,b}-\sum_{i=2}^{5}P^{i}\right)\cdot \max\left[C_{e}\left(z\right),C_{s}\left(z\right)\right] \end{array}$$

where $S_{s}\left(z\right)$ and $C_{s}\left(z\right)$ represent the PGF of $T_{s,suc}$ and $T_{s,col}$, respectively, and the expressions are given in Equation (43). According to Figure 8a, we can use Mason formula to solve the transfer function from the “Start" point to the “End" point, i,e., the PGF of service time of $TS_{e}$. We have

(42) $$P_{TS_{e}}\left(z\right)=\sum_{k=0}^{\infty }q_{e,k}z^{ts_{e,k}}=\frac{S_{e}\left(z\right)}{W_{e}}\sum_{k=0}^{W_{e}-1}{\left[H_{e}\left(z\right)\right]}^{k}$$

In VANETs, nodes use two-way handshake to make SCH reservations. Service provider sends a WSA packet and piggybacks with service information and the selected SCH. Service user responds to the WSA packet with an ACK. The PGF of $T_{s,suc}$ and $T_{s,col}$, $S_{s}\left(z\right)$ and $C_{s}\left(z\right)$ can be given by

(43) $$\left\{\begin{array}{c} S_{s}\left(z\right)=z^{{}^{\left\lfloor \frac{T_{s,suc}}{\sigma }\right\rfloor }}\\ C_{s}\left(z\right)=z^{{}^{\left\lfloor \frac{T_{s,col}}{\sigma }\right\rfloor }} \end{array}\right.$$

The backoff timer will be decremented by one time slot (σ) with probability ($1-P_{s,b}$). The backoff timer will be frozen if the channel is busy due to the following cases: safety packet error with probability $P^{2}$, successful transmission a safety packet with probability $P^{3}$, WSA packet error with probability $P^{4}$, successful transmission a WSA packet with probability $P^{5}$, collision slots from only emergency packets, only WSA packets or both with probability $P_{s,b}-P^{2}-P^{3}-P^{4}-P^{5}$. Therefore, the PGF of the time that the backoff timer of WSA packet decrements by one can be expressed by

(44) $$\begin{array}{cc} H_{s}\left(z\right) & =\left(1-P_{s,b}\right)z+P^{2}C_{e}\left(z\right)+P^{3}S_{e}\left(z\right)+P^{4}C_{s}\left(z\right)\\ & \;+P^{5}S_{s}\left(z\right)+\left(P_{s,b}-\sum_{i=2}^{5}P^{i}\right)\cdot \max\left[C_{e}\left(z\right),C_{s}\left(z\right)\right] \end{array}$$

The backoff process $B_{s,i}\left(z\right)$ at stage *i* of WSA packets can be expressed by

(45) $$B_{s,i}\left(z\right)=\left\{\begin{array}{c} \frac{1}{W_{s,i}}\sum_{k=0}^{W_{s,i}-1}{\left[H_{s}\left(z\right)\right]}^{k},\begin{array}{c} i\leq m^{\prime } \end{array}\\ \frac{1}{W_{s,m^{\prime }}}\sum_{k=0}^{W_{s,m^{\prime }}}{\left[H_{s}\left(z\right)\right]}^{k},m^{\prime }\leq i\leq m \end{array}\right.$$

As we defined before, the random variable $TS_{s}$ is the duration of time taken for a state transition from the start state (when a WSA packet is reaching the head of its queue and beginning to be served) to the end state (being transmitted successfully or discarded after maximum m times retransmission failures). When nodes use WSA packets to make SCH reservations, transmission failure will incur the increasement of backoff stage with doubled CW. The transmission failure includes two cases: transmission collision and transmission bit error. Due to our Assumption 4, these two cases thus have equal time. As shown in Figure 8b, the PGF of $TS_{s}$, $P_{TS_{e}}\left(z\right)$ can be expressed by

(46) $$\begin{array}{cc} P_{TS_{s}}\left(z\right) & =\left(1-P_{s,f}\right)S_{s}\left(z\right)\sum_{i=0}^{m}\left\{{\left[P_{s,f}C_{s}\left(z\right)\right]}^{i}\prod_{j=0}^{i}B_{s,j}\left(z\right)\right\}\\ & \;+{\left(P_{s,f}C_{s}\left(z\right)\right)}^{m+1}\prod_{j=0}^{m}B_{s,j}\left(z\right) \end{array}$$

Finally, based on Equations (42) and (46), we can derive the arbitrary *n*th moment of service time by differentiation. the average service time can be calculated by

(47) $$TS_{i}=\frac{1}{\mu_{i}}=\sum_{k=0}^{\infty }q_{i,k}\cdot (ts_{i,k}\cdot \sigma )=P_{TS_{i}}^{\prime }\left(z\right){|}_{z=1}$$

Now, we can derive the service time distribution and to solve the values of variables $P_{e,emp}$ and $P_{s,emp}$. However, $P_{e,emp}$ and $P_{s,emp}$ depend on the duration of service time. We employ an iterative algorithm to solve $P_{e,emp}$ and $P_{s,emp}$. The detailed iterative steps are as follows:

Step 1: Initialize $P_{e,emp}$ and $P_{s,emp}$ according to the network condition, and assign initial values for them (ranging from 1 to 0), for example, if the network is under the unsaturated condition, it is better to set the value close to 1.
Step 2: Solve the multivariate nonlinear equation to calculate $P_{i,vc}$, $\tau_{i}$, $\eta_{i}$, $P_{i,b}$, $P_{i,f}$ and $P_{i,arr}$ (i∈[e,s]) according to Equations (8), (9), (14), (17), (18), (28) and (30).
Step 3: Calculate the average service time $TS_{e}$ and $TS_{s}$ according to Equations (40)–(47).
Step 4: If $\left(2\lambda_{e}+2\lambda_{s}\right)/\left(\mu_{e}+\mu_{s}\right)\leq 1$, $P_{e,emp}=1-2\lambda_{e}/\left(\mu_{e}+\mu_{s}\right)$, $P_{s,emp}=1-2\lambda_{s}/\left(\mu_{e}+\mu_{s}\right)$, otherwise, $P_{e,emp}=P_{s,emp}=0$.
Step 5: If both $P_{e,emp}$ and $P_{s,emp}$ converge with previous values, then stop the algorithm; otherwise, go to Step 2 with new $P_{e,emp}$ and $P_{s,emp}$.

**Definition 6** **(*Queuing delay*).** *The queuing delay $TQ_{i}$ is the duration from the time instant when an i (i∈[e,s]) packet arrives at the MAC layer queue to the time instant when it becomes the queue head. According to the Pollaczek–Khintchine mean value formula [46] for M/G/1, the expected queuing delay can be derived by*

(48) $$\left\{\begin{array}{cc} TQ_{e} & =\frac{2\lambda_{e}\left[P_{TS_{e}}^{{}^{\prime \prime }}\left(1\right)+P_{TS_{e}}^{{}^{\prime }}\left(1\right)\right]}{2[1-2\lambda_{e}/\left(\mu_{e}+\mu_{s}\right)]}\\ TQ_{s} & =\frac{2\lambda_{s}\left[P_{TS_{s}}^{{}^{\prime \prime }}\left(1\right)+P_{TS_{s}}^{{}^{\prime }}\left(1\right)\right]}{2[1-2\lambda_{s}/\left(\mu_{e}+\mu_{s}\right)]} \end{array}\right.$$

#### 4.4.2. PRR for Safety Packets and WSA Packets

**Definition 7** **(*Packet Reception Rate (PRR)*).** *The PRR is the percentage of packets that successfully received to the number of packets transmitted [39]. To determine that the transmitted packets by the tagged vehicle are successfully received by any other vehicles within its transmission range, the following conditions must be met: (1) no higher ACs in the same node attempt to transmit; (2) when the tagged vehicle is transmitting, no other vehicles within its carrier-sensing range deliver packets at the same time slot; (3) when the tagged vehicle is transmitting, no hidden terminals start to transmit during the vulnerable period $T_{vuln}$; (4) no transmission errors occur during the packet transmission; and (5) no packets are dropped due to exceeding the retransmission limit. Combined with the above conditions, we can calculate the probability of successful reception of safety packets and WSA packets, $PRR_{e}$ and $PRR_{s}$, respectively, as*

(49) $$\left\{\begin{array}{c} \begin{array}{cc} PRR_{e} & =\left(1-P_{e,vc}\right)\times \sum_{k=0}^{\infty }{\left(1-\eta_{total}\right)}^{k}\frac{{\left(N_{cs}-1\right)}^{k}}{k!}e^{-(N_{cs}-1)}\\ & \;\times {\left[\sum_{k=0}^{\infty }{\left(1-\eta_{total}\right)}^{k}\frac{N_{\mathrm{int}}^{k}}{k!}e^{-N_{\mathrm{int}}}\right]}^{T_{vuln}/T_{virt}}\\ & \;\times (1-P_{e,err})\\ & =\left(1-P_{e,vc}\right)\times e^{-\eta_{total}\left(N_{cs}+N_{\mathrm{int}}T_{vuln}/T_{virt}-1\right)}\\ & \;\times (1-P_{e,err})\\ PRR_{s} & =\left(1-P_{s,vc}\right)\times \sum_{k=0}^{\infty }{\left(1-\eta_{total}\right)}^{k}\frac{{\left(N_{cs}-1\right)}^{k}}{k!}e^{-(N_{cs}-1)}\\ & \;\times {\left[\sum_{k=0}^{\infty }{\left(1-\eta_{total}\right)}^{k}\frac{N_{\mathrm{int}}^{k}}{k!}e^{-N_{\mathrm{int}}}\right]}^{T_{vuln}/T_{virt}}\\ & \;\times \left(1-P_{s,err}\right)\times \left(1-P_{s,drop}\right)\\ & =\left(1-P_{s,vc}\right)\times e^{-\eta_{total}\left(N_{cs}+N_{\mathrm{int}}T_{vuln}/T_{virt}-1\right)}\\ & \;\times \left(1-P_{s,err}\right)\times \left(1-P_{s,drop}\right) \end{array} \end{array}\right.$$

#### 4.4.3. Throughput Analysis

In this section, we analyze the throughput of non-safety packets on the SCHs. According to the specification in IEEE 1609.4 standard, a dedicated CCH is used to provide a unique rendezvous for nodes to transmit, gather, and share information such as safety-related messages and SCH reservation information through exchange WSA/ACK messages. The nodes rendezvous on the CCH only during the CCHI. Note, we assume that nodes can determine the starting time of their transmissions on SCHs after their negotiations on the CCH. However, the fixed switch mechanism between CCH and SCH defined in IEEE 1609.4 standard may come with a drawback: When a larger number of nodes is present to communicate with each other under a density vehicular environment, the single CCH can be highly congested and limited CCHI makes nodes having no enough time to performance SCH reservations, and thus CCH becomes a performance bottleneck. Beside, considering the delivery of non-safety packets only during SCHI and the unique feature of multichannel operation in the IEEE 1609.4 standard such as periodical and synchronous fixed switching between CCH and SCH, the throughput of SCHs is thus lower and the SCHs are not fully utilized. We first calculate the number of successful exchange the WSA/ACK packets on the CCH, and then discuss the control channel bottleneck and the SCHs utilization issue.

Let $N_{sch}$ denote the number of SCHs available under the vehicular environments. According to the specification defined in the IEEE 1609.4 standard, we have

(50) $$T_{SYNC}=T_{CCHI}+T_{SCHI}$$

Let $G_{1}$ represent the average number of successful exchange WSA/ACK packets during the CCHI on the CCH. To observe how the length of CCHI affects $G_{1}$ which in turn affects the throughput of SCHs, we define a variable γ to represent the ratio of CCHI to SI, and we call this scheme γ-varying multichannel scheme. Thus, the γ is defined as

(51) $$\gamma =\frac{T_{CCHI}}{T_{SYNC}},0<\gamma <1$$

According to the fixed switch scheme defined in the IEEE 1609.4 standard, γ is equal to 1/2. We derive the expression of $G_{1}$ as

(52) $$\begin{array}{cc} G_{1} & =\frac{T_{CCHI}\times \left(P_{busy}P_{s,suc}^{v}\right)}{T_{virt}}\\ \; & =\frac{\gamma T_{SYNC}\times \left(P_{busy}P_{s,suc}^{v}\right)}{T_{virt}},0<\gamma <1 \end{array}$$

Let $G_{2}$ be the number of non-safety packets which can be transmitted on all $N_{sch}$ SCHs during the SCHI, we have

(53) $$G_{2}=\frac{\left(1-\gamma \right)T_{SYNC}}{T_{data}}N_{sch},0<\gamma <1$$

where $T_{data}$ be the duration of successful transmission of a non-safety packet, and it is calculated by

(54) $$\begin{array}{cc} T_{data} & =DIFS+\frac{H_{PHY}+H_{MAC}+E\left[L_{data}\right]}{R_{d}}\\ & \;+SIFS+T_{ACK}+2\delta \end{array}$$

where DIFS and $E[L_{data}]$ represent the duration of a DIFS and the payload of a non-safety packet. Note, we still use DIFS instead of AIFS for clarity and simplicity when calculating $T_{data}$.

On the one hand, when $G_{1}\leq G_{2}$, it means when a sender and a receiver are performing SCH reservation, there are always free SCHs. Thus, the SCH reservation is successful as long as the transmission of WSA/ACK packet is successful. In other words, a successful transmission of WSA/ACK means a successful transmission of non-safety message. Under this circumstance, the CCH becomes a performance bottleneck and the SCHs are under utilization.

On the other hand, when the available $N_{sch}$ or SCHI is limited, $G_{1}$ is greater than $G_{2}$, and thus the successful transmission of WSA/ACK packets cannot perform successfully transmission of non-safety packets on the SCHs. The throughput of SCHs also decreases. Under this circumstance, the SCHs becomes a performance bottleneck. Therefore, based on the above analysis, the average total throughput of SCHs can be expressed as

(55) $$\begin{array}{cc} S_{data} & =\left\{\begin{array}{c} \begin{array}{ccc} \frac{G_{1}E\left[L_{data}\right]}{T_{SYNC}} & , & G_{1}\leq G_{2}\\ \frac{G_{2}E\left[L_{data}\right]}{T_{SYNC}} & , & G_{1}>G_{2} \end{array} \end{array}\right.\\ & \begin{array}{c} = \end{array}\left\{\begin{array}{c} \begin{array}{ccc} & \frac{\gamma \left(P_{busy}P_{s,suc}^{v}\right)E\left[L_{data}\right]}{T_{virt}}, & G_{1}\leq G_{2}\\ & \frac{\left(1-\gamma \right)N_{sch}E\left[L_{data}\right]}{T_{data}}, & G_{1}>G_{2} \end{array} \end{array}\right. \end{array}$$

Let $S_{data}^{1609.4}$ be the throughput of non-safety packets transmitted on the SCHs in the IEEE 1609.4 standard. Due to the fixed channel switch between CCHI and SCHI defined in the IEEE 1609.4 standard, during a synchronization interval, only half of $T_{SYNC}$ is used to transmit the non-safety packets, and thus $S_{data}^{1609.4}$ can be expressed as Equation (55) when γ is equal to 1/2.

## 5. Model Validation and Numerical Analysis

In this section, the proposed analytical model is applied to a specific VANETs environment for the evaluation of the performance of reliability and effectiveness of the multichannel defined in the IEEE 1609.4 standard, in terms of packet transmission delay, PRR and throughput. We adopt VISSIM [47] and NS-2 simulator [48] for road traffic and network simulations, respectively. Moreover, we implement 802.11e EDCA [49] developed by the TKN group in the Technical University of Berlin. Some parts of original codes have been modified according to the IEEE 802.11p and the IEEE 1609.4 standard.

The simulation scenario is on a 6-km-long highway with one lane in each direction as shown in Figure 4. Every vehicle has a GPS and a single-radio WAVE communication device with parameters shown in Table 4. With regard to ACs assignment to safety-related packets and WSA packets, we refer to [20] to set the values of $W_{e}$, $W_{s,0}$, AIFSN[0] and AIFSN[1]. The simulation scenario description is given in Table 5. All nodes can act as both service providers and service users. The vehicle density is varied from 0.01 to 0.08. Due to the performance analysis in terms of throughput, this model focuses on the effect of fixed switching defined in the IEEE 1609.4 standard on throughput versus the γ-varying multichannel scheme, and thus we set $p_{ber}$ equal to zero on the SCHs.

**Figure 9:**
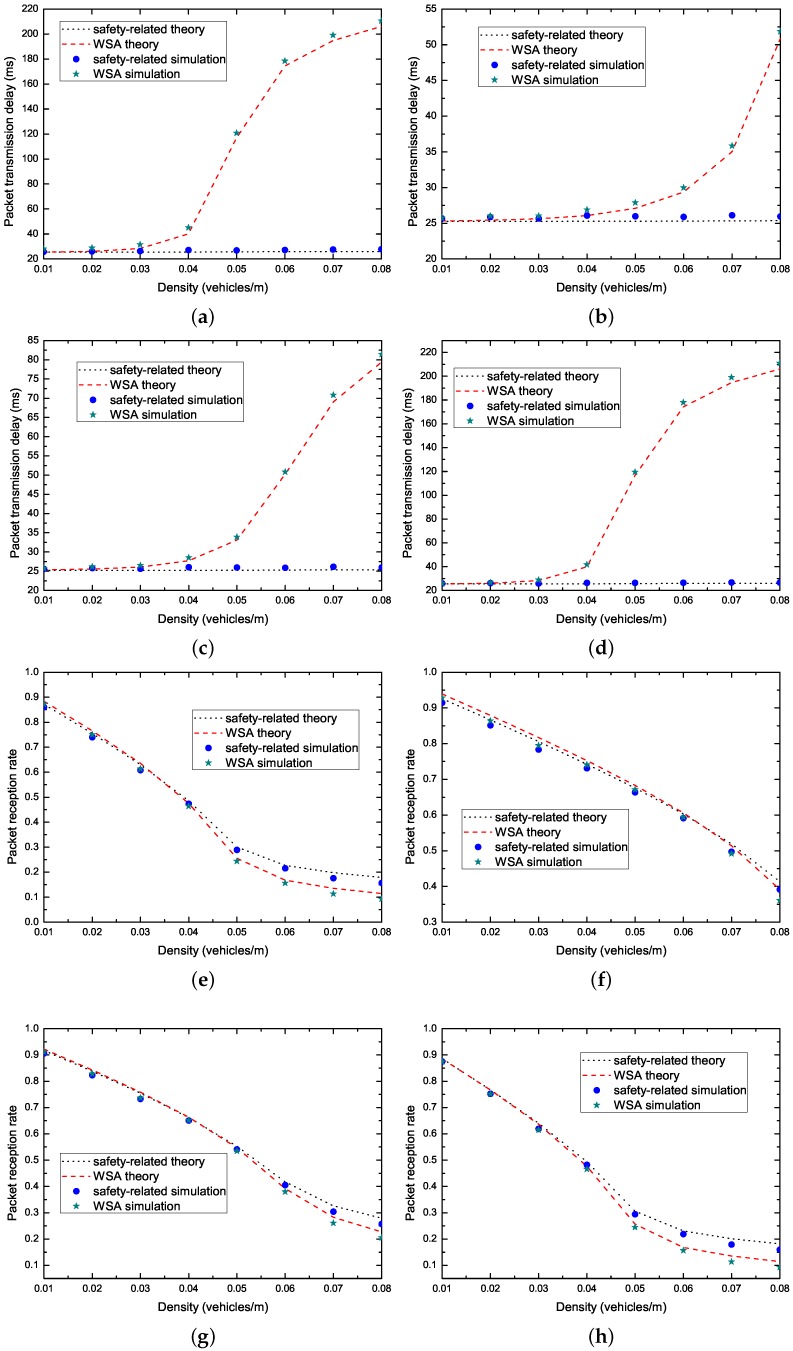

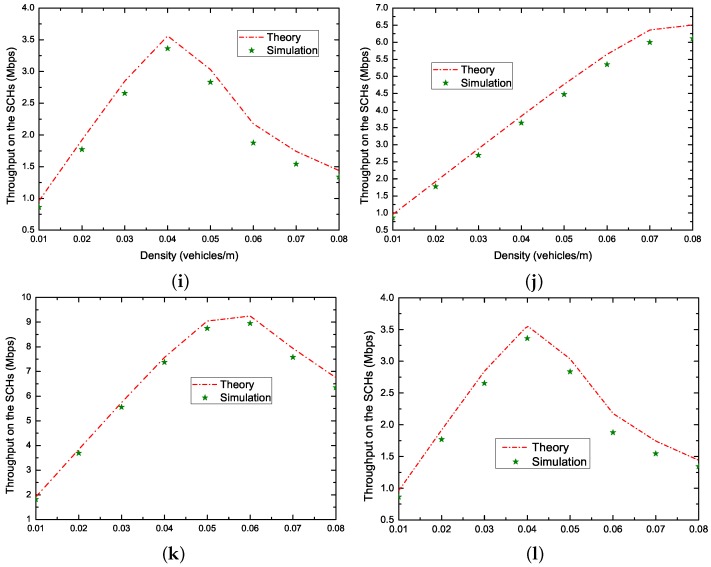
Average packet transmission delay and PRR on the CCH, and throughput on the SCHs: (**a**) packet transmission delay on parameter configurations ➀; (**b**) packet transmission delay on parameter configurations ➁; (**c**) packet transmission delay on parameter configurations ➂; (**d**) packet transmission delay on parameter configurations ➃; (**e**) PRR on parameter configurations ➀; (**f**) PRR on parameter configurations ➁; (**g**) PRR on parameter configurations ➂; (**h**) PRR on parameter configurations ➃; (**i**) throughput on parameter configurations ➀; (**j**) throughput on parameter configurations ➁; (**k**) throughput on parameter configurations ➂; and (**l**) throughput on parameter configurations ➃.

Figure 9 shows the packet transmission delay and PRR on the CCH, and the throughput on the SCHs under varied vehicle density. We change the channel data rate, packet arrival rate, bit error rate and the payload of safety-related packets. The parameter configurations are: ➀ $R_{d}$ = 6 Mbps, $\lambda_{e}=\lambda_{s}$ = 5 packets/s, $p_{ber}=10^{-5}$, $L_{e}$ = 200 Bytes, $L_{s}$ = 160 bits, $L_{data}$ = 2000 Bytes; ➁ $R_{d}$ = 12 Mbps, $\lambda_{e}=\lambda_{s}$ = 5 packets/s, $p_{ber}=10^{-5}$, $L_{e}$ = 200 Bytes, $L_{s}$ = 160 bits, $L_{data}$ = 2000 Bytes; ➂ $R_{d}$ = 12 Mbps, $\lambda_{e}=\lambda_{s}$ = 10 packets/s, $p_{ber}=10^{-5}$, $L_{e}$ = 100 Bytes, $L_{s}$ = 160 bits, $L_{data}$ = 2000 Bytes; and ➃ $R_{d}$ = 6 Mbps, $\lambda_{e}=\lambda_{s}$ = 5 packets/s, $p_{ber}=0$, $L_{e}$ = 200 Bytes, $L_{s}$ = 160 bits, $L_{data}$ = 2000 Bytes. It can be seen that analytical and simulation results match well. According to the results in Figure 9, we can observe that the packet transmission delay for the safety-related messages meets the real-time requirement, however, the PRR for the safety-related messages and the throughput for the non-safety messages rapidly decreases when the vehicle density changes from smooth to jammed.

**Figure 10:**
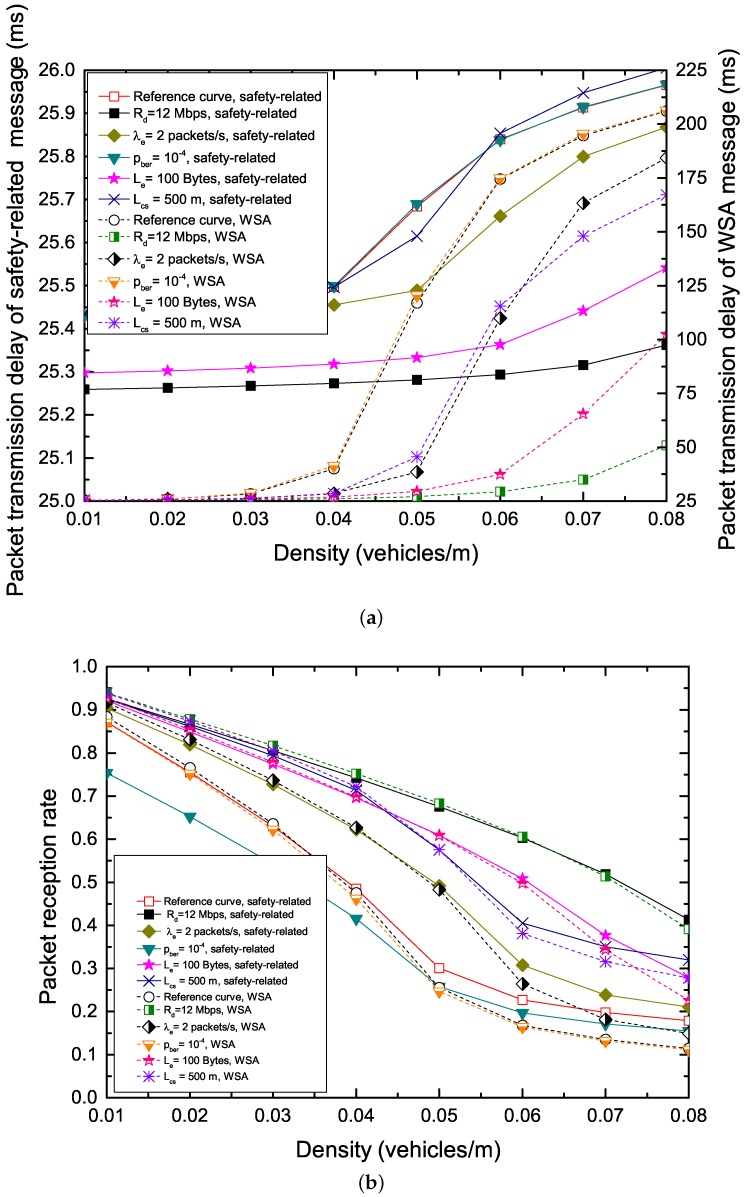

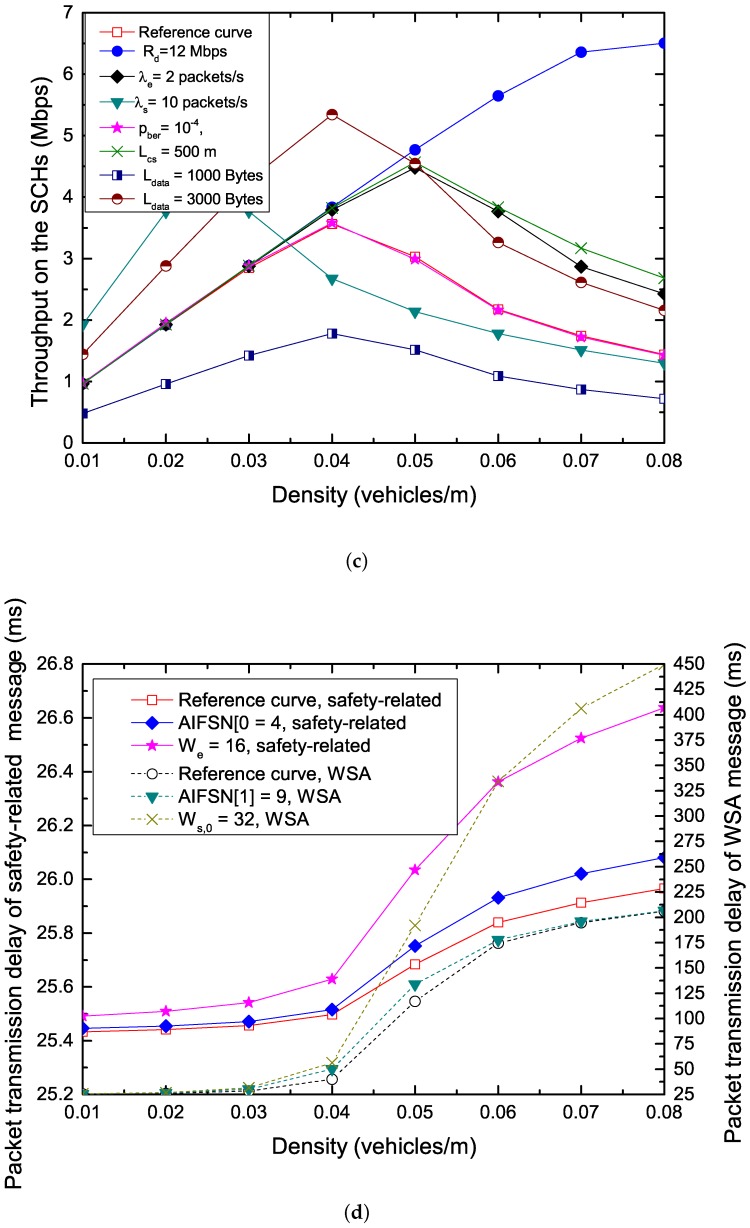

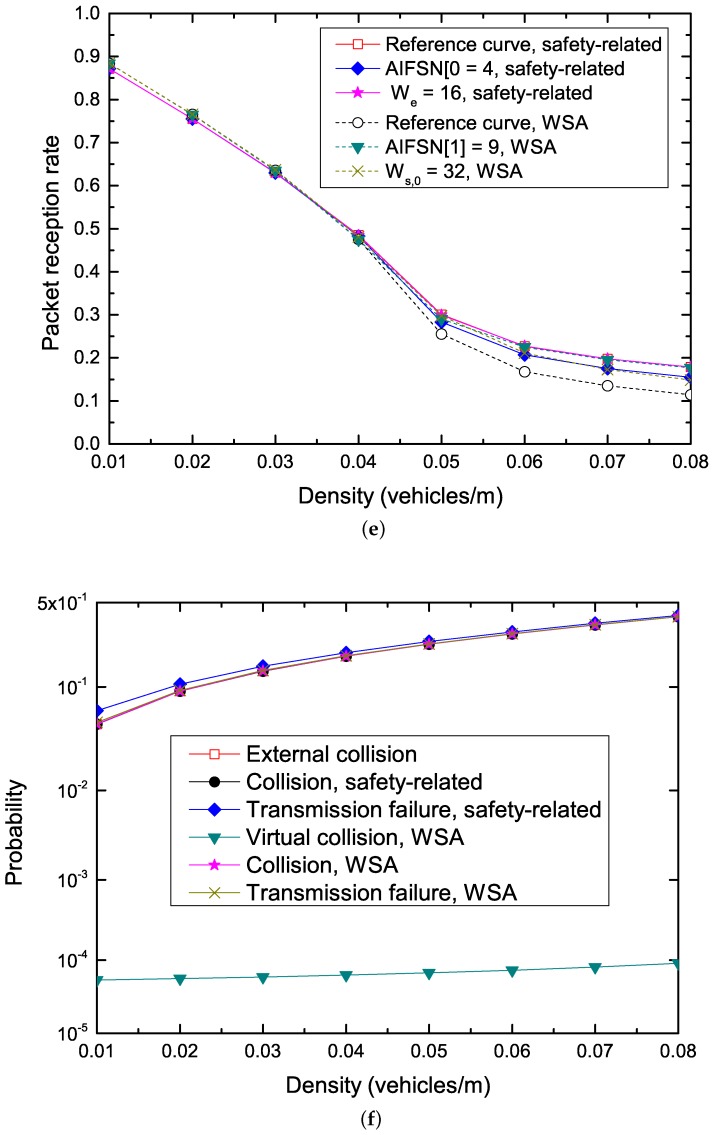

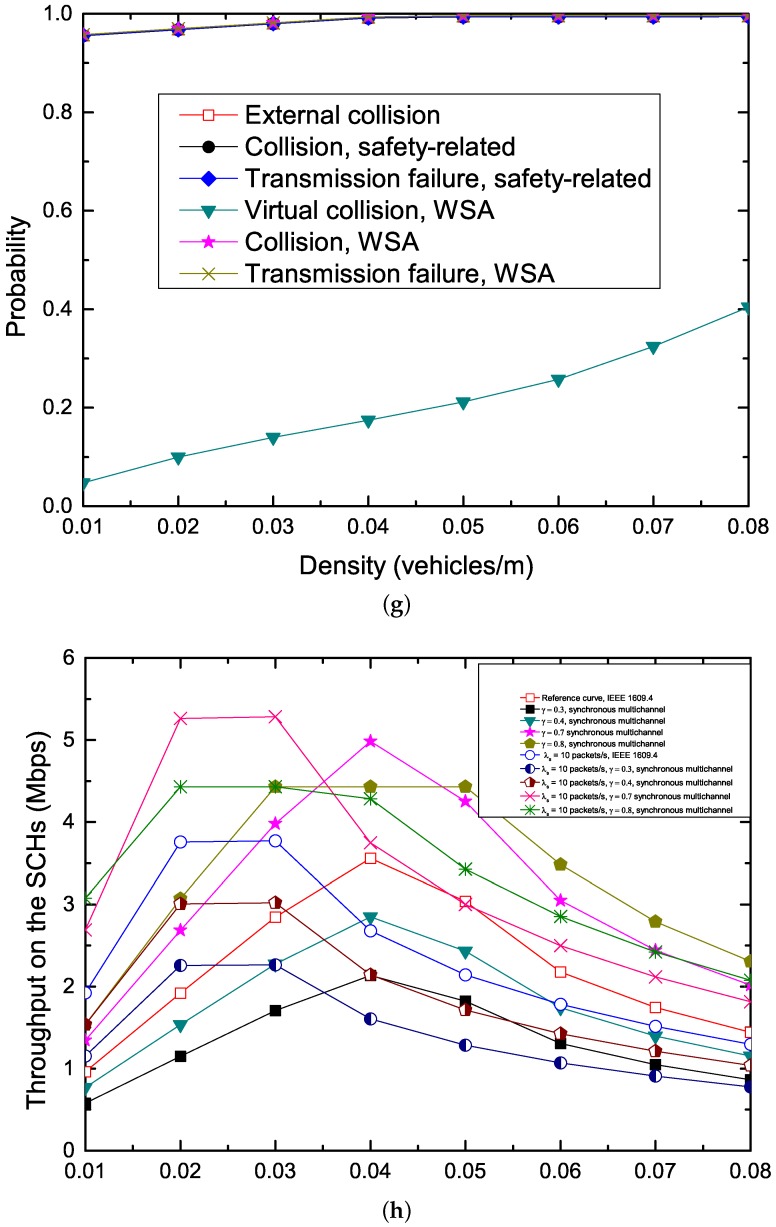
Numerical analysis: (**a**) packet transmission delay of safety-related and WSA message with different parameters; (**b**) PRR of safety-related and WSA message with different parameters; (**c**) throughput on the SCHs: IEEE 1609.4 standard with different parameters of safety-related and WSA message; (**d**) packet transmission delay of safety-related and WSA message with different CW and AIFSN; (**e**) PRR of safety-related and WSA message with different CW and AIFSN; (**f**) comparison of probability of external collision, virtual collision, the collision and transmission failure ($\lambda_{e}=\lambda_{s}$ = 2 packets/s); (**g**) comparison of probability of external collision, virtual collision, the collision and transmission failure ($\lambda_{e}=\lambda_{s}$ = 500 packets/s); and (**h**) comparison of throughput on the SCHs: IEEE 1609.4 standard versus γ-varying multichannel scheme.

Figure 10 analyzes the performance of safety and WSA messages on the CCH and the performance of non-safety messages on the SCHs in detail. The configurations parameters are: $R_{d}$ = 6 Mbps, $\lambda_{e}=\lambda_{s}$ = 5 packets/s, $p_{ber}$ = $10^{-5}$, $L_{e}$ = 200 Bytes, $L_{s}$ = 160 bits, $L_{data}$ = 2000 Bytes, $L_{cs}$ = 400 m, safety-related packet: AIFSN[0] = 3, $W_{e}$ = 8 and WSA packet: AIFSN[1] = 6, $W_{s,0}$ = 16.

Figure 10a–c shows the packet transmission delay and PRR of safety-related and WSA packets with different parameters on the CCH, and the throughput of non-safety packets with different parameters on the SCHs in the IEEE 1609.4 standard. We can observe the following: (1) A higher channel data rate can reduce transmission delay greatly and enhance reliability, at the same time, improve the throughput greatly. On the one hand, this is due to the fact that a higher channel data rate can shorten the packet transmission time, and thus reduce the chances of contentions on the CCH which the conflict probability on the CCH is reduced ultimately. On the other hand, a higher channel data rate can transmit more packets on the SCHs in a short time. (2) With the decrease in the packet arrival rate, the PRR increases significantly, and the packet transmission delay is also reduced. Moreover, the throughput enhances greatly with the decrease of the packet arrival rate. This is due to the fact that the lower packet arrival rate decrease the collision probability and shortens the backoff blocking time, and thus nodes have more opportunities to make SCH reservation and the number of successful reservations rises. (3) Increasing the bit error rate can decrease the PRR sharply, and the throughput also is cut down. This is because the length of a safety-related packet is greater than that of a WSA packet, and thus the effect of bit error rate on the transmissions of safety-related packets is greater than that of WSA packets according to Equation (6). The increase of the bit error rate has no effect on the packet transmission delay. (4) The decrease in the size of safety-related packets has a positive effect on both the packet transmission delay and the reliability. However, the performance gain in terms of throughput increases with the increase of non-safety packet size, and this is because each transmission of non-safety packet with long payload carries more data than that of non-safety packet with short payload. (5) With the increase in the carrier sensing range, both the PRR and the throughput rise significantly, however, the packet transmission delay increases. On the one hand, this is because that the larger carrier sensing range can handle the hidden terminal problem effectively, however, the tagged vehicle also has to supervise more nodes, and thus, leads to a longer service time of packets. On the other hand, the increasing PRR can suppress the packet transmission delay which matter less to the number of successful SCH reservations as the increase of the carrier sensing range, which leads to the increase of throughput on the SCHs ultimately.

Figure 10d,e shows how the CW and AIFSN affect the reliability and performance of the network. We give the reference curves with parameters: safety-related packet: AIFSN[0] = 3, $W_{e}$ = 8 and WSA packet: AIFSN[1] = 6, $W_{s,0}$ = 16, $m^{\prime }$ = 2, *m* = 4. It can be observed that bigger CW size and bigger AIFSN help improve the reliability and also increase the packet transmission delay. This is because that bigger CW size and bigger AIFSN generate fewer contentions and reduce the collision probability, and also increase packet service time.

Figure 10f,g gives the comparison of probabilities of external collision, virtual collision, collision and transmission failure under unsaturated condition ($\lambda_{e}$ = $\lambda_{s}$ = 2 packets/s) and saturated condition ($\lambda_{e}$ = $\lambda_{s}$ = 500 packets/s. Note that the real packets arrival rate for safety traffic and WSA traffic are $2\lambda_{e}$ (1000 packets/s) and $2\lambda_{s}$ (1000 packets/s) on the CCHI, respectively. It can be observed that the virtual collision probabilities are very small under both conditions. Although the safety-related packet has higher priority than WSA packet, the probabilities of collisions of safety-related packets almost are equal to the probabilities of collisions of WSA packets. This is due to that the external collision probability has a primary impact on PRR. In the figure, it can be seen that the transmission failure probabilities of safety-related packets are slightly bigger than that of WSA packets since a safety-related packet size is bigger than that of a WSA packet which incurs the safety-related packet having higher packet error probability. In this case, we need to decrease the packet error probability due to bit error rate, especially in dynamic mobile networks, and this is our future work.

Figure 10h gives the comparison of throughout on the SCH: IEEE 1609.4 standard versus γ-varying multichannel scheme. In order to facilitate the comparison between IEEE 1609.4 standard and γ-varying multichannel scheme, we set $p_{ber}=0$. It can be observed that, in the IEEE 1609.4 standard, the throughput rises at first and then reduces (or keep maximum values for a small segment length and then reduces) when the vehicle density rises further. The reason is that, taking $\lambda_{e}$ = $\lambda_{s}$ = 5 packets/s for example, when the vehicle density is less than 0.04 vehicles/m, due to the longer CCHI and the shortage of WSA packets, the CCH is idle for a while, and then the throughput is lower. When the vehicle density is more than 0.04 vehicles/m, higher vehicle density generates more contentions and requires more CCHI to make SCH reservations. Under the above two conditions, the SCHs are starved state, and the SCHs is under utilized. From Figure 10h, it can be seen when the ratio of CCHI to SI, γ, increases within the range of 0.7, the maximum throughput vales of γ-varying multichannel scheme also increases. While when γ increases from 0.7 to 1, the throughput of γ-varying multichannel scheme deceases. This is because that, when γ≤ 0.7, due to the effect of factors such as virtual collision, channel fading and hidden terminals, the number ($G_{1}$ in Equation (52)) of successful SCH reservations is smaller, and then the SCHs are idle for a long time. Therefore, the SCH throughput is lower and the SCHs are under utilized, and the CCH is the performance bottleneck. On the other hand, when γ > 0.7, due to the shortage of SCHI, the number ($G_{2}$ in Equation (53)) of non-safety packets which can be allowed to be transmitted on the SCHs is smaller ($G_{2}$ < $G_{1}$). Under this condition, SCH is the performance bottleneck, and the CCH is under utilized. In these cases, we need to determine the γ dynamically based on the vehicle density so as to obtain an efficient and optimal throughput.

## 6. Conclusions and Future Work

In this paper, we proposed two Markov chain models for safety-related messages and WSA messages to analyze the real-time and reliability of broadcasting of safety-related messages on CCH and to analyze the efficiency of throughput and utilization of SCHs under both unsaturated and saturated conditions. We analyze in detail packet transmission delay, PRR and throughput by considering virtual collision, AIFS differentiation, CW, the retry limit, frame blocking probability, channel busy probability, frame failure probability and channel switch. We employ M/G/1 queuing model to derive a more accurate analytical result.

By simulations, we validate the proposed models, and the analytical and simulation results match well. From the proposed analytical models and the numerical results, in addition to some straightforward observations, we obtained several important conclusions: (1) Under vehicular environments, IEEE 1609.4 standard is able to meet the transmission delay requirement of safety-related messages, however, it is difficult to meet the reliability requirement due to the transmission collision and harsh channel fading. (2) Hidden terminal problem is the main factor affecting the external collision which degrades the PRR significantly. (3) High channel data rate leads to higher reliability for transmitting both safety-related and WSA packets. (4) IEEE 1609.4 standard results in inefficient utilization of both CCH and SCH, i.e., utilization cannot inherently exceed 50%. (5) The fixed channel switching between CCH interval and SCH interval prohibits adaptive and intelligent allocation of time interval in response to variable traffic demands, and thus, leads to inefficiency throughput and underutilization of SCHs. (6) On the one hand, the potential ways to improve reliability for broadcast safety-related messages on the CCH include using bigger CW to reduce the possibility of concurrent transmissions, choosing larger carrier-sensing range to mitigate the hidden terminal problem, increasing the transmission data rate, increasing the number of packet retransmission, etc. On the other hand, the potential ways to improve the throughput and utilization of SCHs include employing the higher transmission data rate, increasing the number of SCHs, using the larger non-safety packet payload to carry more data, increasing the SCH interval, providing the contention-free transmission, etc.

In our future research, we will focus on how to ensure the reliable transmission of safety-related messages, and improve the throughput and utilization of SCHs by adjusting system parameters dynamically. we will also exploit an efficient method to solve the hidden terminal problem, and explore appropriate method to decrease the BER caused by fading channel. Finally, the proposed model performance under real field test will be considered. 

## Figures and Tables

**Table 1:** Comparison of different models.

Model	Protocol	MAC Type	Markov Chain	H/E	BBP	BSIP	AD	VC	CS	Network Condition	Traffic Type	D, T PRR	Year
Bianchi [29]	802.11a	DCF	2-D	H/×	Equal to 1	FCP	–	–	×	S	U	×, T/×	2000
Wang et al. [24,26]	802.11p	DCF	–	×/×	–	–	–	–	√	NS	B/U	×,×/PRR	2008
Ma et al. [14]	802.11p	DCF	1-D	H/×	Equal to 1	–	–	–	×	NS	B	×, ×/PRR	2011
Misic et al. [27]	802.11p	EDCA	2-D	×/E	FBP	FFP	√	√	√	NS/S	U	D, ×/×	2011
Hafeez et al. [15,16]	802.11p	EDCA	1-D	H/E	FCP	–	√	×	×	NS	B	D, T/PRR	2013
Yao et al. [17,18]	802.11p	EDCA	1-D/2-D	H/×	FBP	FVCP	√	√	×	NS/S	B	D, ×/PRR	2013
Campolo et al. [19,20,22]	802.11p	DCF	–	×/×	–	–	–	–	√	NS	B	×, ×/PRR	2013
Yin et al. [21]	802.11p	DCF	–	H/E	CBP	–	–	–	√	NS	B	D, ×/PRR	2014
Xiong et al. [28]	802.11p	EDCA	1-D/2-D	H/E	CBP	FVCP	×	×	√	NS/S	B/U	×, T/PRR	2015
Proposed Model	802.11p	EDCA	1-D/2-D	H/E	FBP	FFP	√	√	√	NS/S	B/U	D, T/PRR	2017

Note: H/E: Hidden terminal/Erroneous channel, BBP: Backoff Blocking Probability, BSIP: Backoff Stage Increase Probability, AD: AIFS Differentiation, VC: Virtual Collision, CS: Channel Switch, D: Delay, T: Throughput, PRR: Packet Reception Rate, NS/S: Non-saturation/Saturation, B/U: Broadcast/Unicast, FCP: Frame Collision Probability, FBP: Frame Blocking Probability, FVCP: Frame Virtual Collision Probability, FFP: Frame Failure Probability, CBP: Channel Busy Probability.

**Table 2:** Default EDCA parameters in IEEE 802.11p.

AC	CWmin	CWmax	AIFSN[AC]
3	CWmin	CWmax	9
2	CWmin	CWmax	6
1	(CWmin + 1)/2 − 1	CWmin	3
0	(CWmin + 1)/4 − 1	(CWmin + 1)/2 − 1	2

**Table 3:** Summary of important symbols.

Symbol	Definition
β	The vehicle density on the highway (vehicles/m).
*R*	The transmission range of a node.
$L_{cs}$	The carrier-sensing range of a node.
$L_{int}$	The interfering range of a node.
$N_{tr}$	The number of nodes within the transmission/receiving range of tagged node.
$N_{cs}$	The number of nodes within the carrier-sensing range of tagged node.
$N_{int}$	The number of nodes within the interfering range of tagged node.
P(j,l)	The probability that *j* vehicles exist within length *l* of the highway.
$P_{i,arr}$	The arrival probability of *i* packets.
$P_{i,emp}$	The probability that the type of *i* queue is empty.
$\rho_{i}$	The probability that at least one packet waits in the type of *i* queue.
$P_{i,suc}$	The probability that a transmission attempt of an *i* packet is successful.
$P_{i,b}$	The backoff blocking probability that other nodes or other ACs in the same node is occupying the channel.
$P_{i,f}$	The probability that transmission of an *i* packet is failing.
$P_{oc}$	The external collision probability that an *i* packet collides with other nodes while accessing the channel.
$P_{i,vc}$	The virtual collision probability that an *i* packet collides with the higher priorities inside a node.
$P_{i,c}$	The collision probability of an *i* packet.
$P_{i,err}$	The *i* packet error probability caused by channel fading.
$p_{ber}$	The bit error rate.
$\tau_{i}$	The internal transmission probability that the probability of transmission attempt for access class *i* in a random time slot observed by the other ACs of the same station.
$\eta_{i}$	The external transmission probability that the probability of transmission attempt for access class *i* observed by other node.
$\eta_{total}$	The total transmission probability of a station.
$P_{s,drop}$	The probability that a WSA packet is dropped due to more than retransmission limit.
$P_{busy}$	The probability that the channel is busy in a time slot.
$P_{i,suc}^{v}$	The probability that the transmission attempt of an *i* packet is successful, conditioned on the fact that at least one station transmits in the considered time slot.
$P_{col}$	The probability that a transmission fails due to a collision given that there is at least one station transmits in the considered time slot.
$PRR_{i}$	The packet reception rate is the percentage of packets that successfully received to the number of packets transmitted.
$T_{i,suc}$	The duration for successful transmitting an *i* packet.
$T_{i,col}$	The duration for a transmission collision caused by *i* traffic.
AIFS[*i*]	The duration of AIFS of *i* traffic.
SIFS	The duration of an SIFS.
$m^{\prime }$	The maximum times the CW can be doubled.
*m*	The maximum retransmission number.
$M_{ln}$	The number of lanes in each direction on the highway.
δ	The propagation delay.
σ	A time slot.
$T_{vuln}$	Vulnerable period during which the transmission of the tagged node may be subjected to hidden terminal interference.
$T_{virt}$	The average duration of a virtual time slot.
$T_{SYNC}$	The duration of a synchronization interval.
$T_{CCHI}$	The duration of CCH interval.
$T_{SCHI}$	The duration of SCH interval.
$T_{i}$	The transmission delay of an *i* packet.
$T_{i,suc}$	The time of successful transmission of an *i* packet.
$T_{i,col}$	The time of transmission collision of an *i* packet.
$T_{ACK}$	The time for transmitting an ACK packet.
$H_{MAC}$	The header length of MAC-layer of a packet.
$H_{PHY}$	The header length of physical-layer of a packet.
$L_{i}$	The payload of an *i* packet.
$L_{ACK}$	The packet size of an ACK packet.
$R_{d}$	The transmission data rate on the CCH and SCH.
$W_{e}$	The CW size for safety packets.
$W_{s,i}$	The CW size for WSA packets in the *i*th backoff stage.
$b_{I_{i}}$	The stationary distribution of idle state for *i* traffic.
$b_{e,k}$	The stationary distribution of backoff state *k* for safety traffic.
$b_{s,i,k}$	The stationary distribution of the backoff state *k* in stage *i* for WSA traffic.
$\frac{1}{\mu_{i}}$	The average service time of *i* packets.
$TQ_{i}$	The queuing delay that the duration from the time instant when an *i* packet arrivals at the MAC layer queue to the time instant when it becomes the head of queue.
$TS_{i}$	The service time is the duration from the time instant when an *i* packet becomes the head of the queue to the time instant when this packet is transmitted or dropped.
$D_{i}$	The transmission delay is the time duration between the time instant that an *i* packet arrives at the queue to the time instant that this packet is successful transmitted or dropped.
γ	The ratio of CCHI to SI.
$N_{sch}$	The number of available SCHs in VAENTs.
$G_{1}$	The average number of successful SCHs reservation made on CCH.
$G_{2}$	The number of non-safety packets transmitted on all $N_{sch}$ SCHs.
$S_{data}$	The average total throughput of SCHs.

Note: *i* except $W_{s,i}$ and $b_{s,i,k}$ stands for *e* and *s* which means emergency (safety) and WSA, respectively.

**Table 4:** Parameter for communications in DSRC.

Parameter	Value
Frequency	5.9 GHz
Modulation	QPSK, 16QAM
Signal bandwidth	10 MHz
Channel data rate ($R_{d}$)	6, 12 Mbps
$W_{e}$	8
$W_{s,0}$	16
AIFSN[0]	3
AIFSN[1]	6
$m^{\prime }$	2
Retry limit (*m*)	4
SIFS	32 μs
DIFS	64 μs
Slot time (σ)	13 μs
Propagation delay (δ)	1 μs

**Table 5:** Parameter for road traffic.

Paramenter	Value
Highway length	6000 m
Number of lanes	1 lane in each direction
Vehicle density (β)	0.01 to 0.08 vehicles/m
Transmission range (*R*)	300 m
Carrier sensing range ($L_{cs}$)	400 m
Packet arrival rate ($\lambda_{e}$), ($\lambda_{s}$)	5 packets/s
The bit error rate ($p_{ber}$)	$10^{-5}$
MAC-layer header length ($H_{MAC}$)	256 bits
Physical-layer header length ($H_{PHY}$)	192 bits
An ACK packet size ($L_{ACK}$)	$H_{PHY}$ + 112 bits
The payload of a safety packet ($L_{e}$)	200 Bytes
The payload of a WSA packet ($L_{s}$)	160 bits
The payload of a non-safety packet ($L_{data}$)	2000 Bytes
